# Activin receptors regulate the oligodendrocyte lineage in health and disease

**DOI:** 10.1007/s00401-018-1813-3

**Published:** 2018-02-03

**Authors:** Alessandra Dillenburg, Graeme Ireland, Rebecca K. Holloway, Claire L. Davies, Frances L. Evans, Matthew Swire, Marie E. Bechler, Daniel Soong, Tracy J. Yuen, Gloria H. Su, Julie-Clare Becher, Colin Smith, Anna Williams, Veronique E. Miron

**Affiliations:** 1 Medical Research Council Centre for Reproductive Health, The Queen’s Medical Research Institute, The University of Edinburgh, 47 Little France Crescent, Edinburgh, EH16 4TJ UK; 2 Medical Research Council Centre for Regenerative Medicine, The University of Edinburgh, 5 Little France Drive, Edinburgh, EH16 5UU UK; 3 Department of Veterinary Medicine, Wellcome Trust MRC Cambridge Stem Cell Institute, University of Cambridge, Cambridge, CB3 0E5 UK; 4 Department of Pathology and Cell Biology, Herbert Irving Comprehensive Cancer Center, Columbia University Medical Centre, New York, 10032 USA; 5 Simpson’s Centre for Reproductive Health, Royal Infirmary of Edinburgh, Edinburgh, EH16 4SA UK; 6 Centre for Clinical Brain Sciences, Centre for Comparative Pathology, The University of Edinburgh, Chancellor’s Building, Edinburgh, EH16 4TJ UK; 7 Present Address: Department of Neuroscience, Genentech Inc., South San Francisco, CA 94080 USA

**Keywords:** Myelin, Remyelination, Oligodendrocyte, Multiple sclerosis, Perinatal brain injury, Activin receptor

## Abstract

The most prevalent neurological disorders of myelin include perinatal brain injury leading to cerebral palsy in infants and multiple sclerosis in adults. Although these disorders have distinct etiologies, they share a common neuropathological feature of failed progenitor differentiation into myelin-producing oligodendrocytes and lack of myelin, for which there is an unmet clinical need. Here, we reveal that a molecular pathology common to both disorders is dysregulation of activin receptors and that activin receptor signaling is required for the majority of myelin generation in development and following injury. Using a constitutive conditional knockout of all activin receptor signaling in oligodendrocyte lineage cells, we discovered this signaling to be required for myelination via regulation of oligodendrocyte differentiation and myelin compaction. These processes were found to be dependent on the activin receptor subtype Acvr2a, which is expressed during oligodendrocyte differentiation and axonal ensheathment in development and following myelin injury. During efficient myelin regeneration, Acvr2a upregulation was seen to coincide with downregulation of Acvr2b, a receptor subtype with relatively higher ligand affinity; Acvr2b was shown to be dispensable for activin receptor-driven oligodendrocyte differentiation and its overexpression was sufficient to impair the abovementioned ligand-driven responses. In actively myelinating or remyelinating areas of human perinatal brain injury and multiple sclerosis tissue, respectively, oligodendrocyte lineage cells expressing Acvr2a outnumbered those expressing Acvr2b, whereas in non-repairing lesions Acvr2b+ cells were increased. Thus, we propose that following human white matter injury, this increase in Acvr2b expression would sequester ligand and consequently impair Acvr2a-driven oligodendrocyte differentiation and myelin formation. Our results demonstrate dysregulated activin receptor signaling in common myelin disorders and reveal Acvr2a as a novel therapeutic target for myelin generation following injury across the lifespan.

**Electronic supplementary material:**

The online version of this article (10.1007/s00401-018-1813-3) contains supplementary material, which is available to authorized users.

## Introduction

Myelin ensures axon health and function in the CNS via trophic/metabolic support and insulation for electrical impulse conduction [14, 16, 26, 32, 37, 46]. The lack of myelin is, therefore, associated with axon dysfunction and/or loss, causing deficits in movement, sensation, and cognition, as observed in prevalent myelin disorders of development (perinatal brain injury leading to cerebral palsy/cognitive deficits) and adulthood [multiple sclerosis (MS)]. Although these have distinct etiologies, a shared neuropathological feature involves failed differentiation of oligodendrocyte precursor cells (OPCs) into myelin-producing oligodendrocytes following injury, resulting in impaired myelin formation (myelination) [8, 10, 33, 36, 57] or regeneration (remyelination) [38], respectively. However, the mechanisms underpinning this pathology are not fully understood, as evidenced by the lack of approved therapies aimed at promoting oligodendrocyte differentiation and myelin generation.

Our previous work identified activin-A, a member of the transforming growth factor beta (TGF-β) superfamily, as being a promising pro-differentiation therapeutic target [45]. We found that at the onset of remyelination of focally demyelinated white matter lesions, microglia/macrophages express activin-A, and oligodendrocyte lineage cells express the ligand-binding activin receptors (Acvr2a, Acvr2b) and the signal-transducing co-receptor (Acvr1b) [45]; depletion of these microglia/macrophages caused impairment of remyelination [45]. In addition, healthy developmental myelination has recently been shown to be regulated by other TGF-β family members, TGF-β1 and activin-B [19, 49], albeit primarily via signaling through distinct receptors (TGFβR1) or co-receptors (Acvr1c), respectively. However, how activin receptor signaling regulates oligodendroglial lineage cell behavior, and whether this is required for myelination and remyelination, remains to be fully elucidated.

Here, we reveal the requirement for activin receptor signaling in regulating oligodendrocyte lineage cell responses during healthy white matter development and following injury. Furthermore, we demonstrate how dysregulation of activin receptor expression underpins myelin pathology in human perinatal brain injury and multiple sclerosis, revealing potentially targetable receptors for clinical intervention in myelin disorders across the lifespan.

## Materials and methods

### Animals

All experiments were performed under UK Home Office project licenses issued under the Animals (Scientific Procedures) Act. Animals were housed at 6 animals per cage in a 12 h light/dark cycle with unrestricted access to food and water. For animal experiments, power was calculated by two-sided 95% confidence interval via the normal approximation method using OpenEpi software, and reached > 80% power (84–100%) for all experiments. *ARRIVE* guidelines were followed in providing details of experiments, quantifications, and reporting.

### Organotypic cerebellar slice cultures

Postnatal day 0–2 (P0-P2) CD1 pup cerebellum and attached hindbrains were sagittally sectioned at 300 µm on a McIlwain tissue chopper and plated onto Millipore-Millicel-CM mesh inserts (Fisher Scientific) in 6-well culture plates at six slices per insert. Media was composed of 50% minimal essential media, 25% heat-inactivated horse serum, 25% Earle’s balanced salt solution (all from GIBCO), 6.5 mg ml^−1^ glucose (Sigma), 1% penicillin–streptomycin, and 1% glutamax. At 21 days in vitro when myelination is complete and compact, demyelination was induced by incubation in 0.5 mg ml^−1^ lysolecithin (Sigma) for 18-20 h. Slices were then washed in media for 10 min and treated at 2 days post lysolecithin (dpl) until 7, 10, or 14 dpl with activin-A (100 ng ml^−1^, R&D Systems), inhibin-A (100 ng ml^−1^, R&D Systems) or vehicle controls. Slices were fixed in 4% paraformaldehyde (PFA, wt/vol) for 10 min and blocked in 5% normal horse serum (GIBCO) and 0.3% Triton-X-100 (Fisher Scientific) for 1 h. Primary antibodies rat anti-MBP (1:250, AbD Serotec; MCA409S) and chicken anti-neurofilament-H (1:10,000, EnCor Biotech; CPCA-NF-H) were applied for 48 h at 4 °C. Slices were washed twice in 0.1% Triton-X-100 and fluorescently conjugated antibodies applied for 2 h at 20–25 °C (Life Technologies-Molecular Probes). Following counterstaining with Hoechst, slices were washed thrice and mounted onto glass slides using Fluoromount-G. Z-stacks were captured using an Olympus 3i Spinning Disk microscope (60× silicone objective) and SlideBook software. Stacks were cropped to 14 slices (0.59 µm/slice) in SlideBook (3i), and images blinded and imported into Volocity (Perkin Elmer) as an image sequence. Remyelination index was calculated by normalizing voxel counts of values of co-localization of myelin (MBP) and axon (NF) to NF voxel counts, and this value for treated slices was further normalized to vehicle controls. Both males and females were assessed.

### Breeding strategy for conditional knockout generation

Sperm from *Acvr1b* LoxP mice was generously provided by Dr. Gloria H. Su (Columbia University) where exons 2–3 are flanked with Cre-LoxP sites, which upon Cre recombination causes deletion of a 3.3-kb sequence, frameshift mutation, and abolishment of Acvr1b protein expression [53]. Sperms were injected into pseudopregnant C57Bl/6J females. The offspring were intercrossed to generate mice homozygous for the LoxP allele and subsequently crossed to PDGFRa-Cre mice (Jax laboratories, 013148). Mice identified as being positive for PDGFRa-Cre and heterozygous for the LoxP allele were then crossed back to homozygous *Acvr1b* LoxP mice to generate homozygous conditional knockout (cKO) mice. Mice were confirmed as a cKO by performing PCR on the genomic DNA for detection of the Cre recombinase gene and homozygosity of the *Acvr1b* LoxP allele. Further analysis of the recombination by PCR and Cre recombinase immunohistochemistry in the corpus callosum confirmed the conditional status of these mice (Online Resource Supplemental Fig. 1). This was confirmed by DNA extraction from cortical OPCs of transgenic mice using the Wizard SV genomic purification system (Promega) and PCR using Q5 High Fidelity DNA Polymerase (New England Biolabs) using amplification with primers P4 and P5 (sequence in genotyping section below) (Online Resource Supplemental Fig. 1). Both males and females were assessed.

### Genotyping

Genomic DNA was extracted from ear tissue using the Wizard SV genomic purification system (Promega) according to the manufacturer’s instructions. *Acvr1b* floxed mice were genotyped using PCR strategies as previously described [53]. Briefly, *Acvr1b* floxed mice were genotyped using primers P1 (ATGAAAAGTGCTTGCGTGTG) and P2 (CAGGGAAGGGCAGATATCAA). PDGFRa-Cre mice were genotyped using primers 1084 (GCGGTCTGGCAGTAAAAACTATC), 1085 (GTGAAACAGCATTGCTGTCACTT), 7338 (CTAGGCCACAGAATTGAAAGATCT) and 7339 (GTAGGTGGAAATTCTAGCATCATCC). Cre-mediated recombination was detected using P4 (CCAGCACCTACATCACATGG) and P5 (CTCTATGGAGAGCACCCTCTTTG) (Online Resource Supplemental Fig. 1).

### Immunohistochemistry of rodent cryosections

Animals were intracardially perfused with 4% PFA (wt/vol; Sigma), brains post-fixed overnight and cryoprotected in sucrose prior to embedding in OCT (Tissue-Tech) and storage at − 80 °C. 8–10 μm cryosections were air-dried, permeabilized and blocked for 1 h with 5% normal horse serum (GIBCO) and 0.3% Triton-X-100 (Fisher Scientific). For Caspr staining, blocking solution and antibody diluent was composed of 10% normal horse serum and 0.1% Triton-X-100. For myelin protein staining, sections were permeabilized in methanol at − 20 °C for 10 min. For Olig1 and Olig2 staining, sections were permeabilized in Vector Unmasking Solution (Vector) by microwaving for 5 min followed by a 30-min incubation at 60 °C. Primary antibodies were applied overnight at 4 °C in a humid chamber and include rat anti-MBP (AbD Serotec, 1:250; MCA409S), mouse anti-MAG (Millipore, 1:100; MAB1567), mouse anti-Olig2 (Millipore, 1:100; MABN50), rabbit anti-Olig2 (1:100, Millipore; AB960), mouse anti-Olig1 (Millipore, 1:100; MAB5540), mouse anti-NG2 (1:200, Millipore; MAB5384), mouse anti-CC1 (1:100, Abcam; ab16794), rabbit anti-Caspr (1:500, Abcam; ab34151), rabbit anti-cleaved caspase-3 (1:500, BD Pharmingen; 559565), rabbit anti-Acvr2a (1:100, Abcam; ab135634), goat anti-Acvr2a (1:40, RnD Biosystems; AF340), rabbit anti-Acvr2b (1:100, Abgent; AP7105a). Fluorescently conjugated secondary antibodies were applied for 2 h at 20–25 °C in a humid chamber (1:500, Life Technologies-Molecular Probes). Following counterstaining with Hoechst, slides were coverslipped with Fluoromount-G (Southern Biotech). Antibody isotype controls added to sections at the same final concentration as the respective primary antibodies showed little or no nonspecific staining. Terminal deoxynucleotidyl transferase dUTP nick end labeling (TUNEL; Promega) assay for apoptosis was carried out according to the manufacturer’s instructions; DNase I (10 units ml^−1^) was applied as a positive control for double stranded breaks. Sections were imaged on a Leica SPE confocal microscope (40× objective) or an Olympus 3i Spinning Disk microscope (30×, 60×, or 100× oil immersion objectives). Intensity of MAG or MBP staining in selected white matter areas was determined using the Histogram function in Adobe Photoshop and normalized to background levels in a non-white matter area from the respective sample. Percentage area of NF co-localizing with Caspr was calculated using Volocity software.

### Western blotting

CNS samples were lysed with RIPA buffer (Thermo Scientific) supplemented with 1% protease inhibitor cocktail set III ethylenediaminetetraaceticacid (EDTA)-free (Calbiochem). Protein concentrations were determined using the Pierce BCA Protein Assay Kit (according to the manufacturer’s instructions). Samples were diluted in Laemmli buffer (BioRad) and 5% β-mercaptoethanol (Sigma)], heated at 95 °C for 2 min, and 10 µg of protein was loaded onto an acrylamide gel (4–20%; Thermo Scientific). Gel electrophoresis was performed in tris-hydroxyethyl piperazineethanesulfonic acid (HEPES)-sodium dodecyl sulfate (SDS) running buffer (Thermo Scientific) at 100 V and proteins transferred onto polyvinylidene difluoride (PVDF) membranes (Millipore) for 2 h at 10 V in 10% transfer buffer [3% Tris–HCl (Sigma-Aldrich), 15% glycine (Sigma-Aldrich), pH 8.3] and 20% methanol (Fisher Chemical) diluted in water. Membranes were blocked with 4% bovine serum albumin in Tris-buffered saline (TBST) [4% sodium chloride (NaCl), 0.1% potassium chloride (KCl), 1.5% Tris–HCl, 0.1% Tween-20 (all from Sigma-Aldrich), pH 7.4] for 1 h at room temperature on an orbital shaker, then incubated overnight at 4 °C with rabbit anti-MBP (1:1000, AB980; Merck Millipore) or mouse anti-CNPase (1:1000, AMAb91068; Atlas Antibodies). Membranes were washed thrice in TBST for 5 min and incubated with horseradish peroxidase (HRP)-IgG secondary antibody conjugates (1:2000; Calbiochem) for 1 h at room temperature. Chemiluminescent substrate detection reagent RapidStep ECL Reagent (Calbiochem) was used to visualize bands. For loading control purposes, all membranes were re-blotted with anti-mouse or anti-rabbit beta-Actin.

### Resin embedding, semi-thin sections, and electron microscopy

Mice were intracardially perfused with 4% PFA (wt/vol) and 2% glutaraldehyde (vol/vol; TAAB Laboratories) in 0.1 M phosphate buffer. Tissue was post-fixed overnight at 4 °C and transferred to 1% glutaraldehyde (vol/vol) until embedding. 1 mm tissue sections were processed into araldite resin blocks. 1 μm microtome-cut sections were stained with a 1% toluidine blue/2% sodium borate solution prior to bright field imaging at 100× magnification using a Zeiss Axio microscope. Number of myelinated axons was blindly quantified in 50 μm × 50 μm images of corpus callosum, with 2–4 sections counted per mouse and then values averaged. Ultrathin sections (60 nm) were cut from corpus callosum, stained in uranyl acetate and lead citrate, and grids imaged on a JEOL Transmission Electron Microscope. Axon diameter, myelin thickness, and inner tongue thickness were calculated from measured area based on assumption of circularity using Fiji/ImageJ (Fiji.sc) (diameter = 2 × √[area/π]), with a minimum of 100 axons per animal analyzed. Enlarged inner tongues in conditional knockout mice precluded standard *g*-ratio analysis of myelin thickness, which was instead calculated by subtracting the diameter to the innermost compact myelin layer from the diameter to the outermost compact myelin layer (see Online Resource Supplemental Fig. 5 for outline of quantification methods). Inner tongue thickness was calculated by subtracting the axonal diameter from the diameter to the innermost compact layer (Online Resource Supplemental Fig. 5). ‘Proportion of myelinated axons’ per axon diameter was determined by plotting the proportion of total myelinated axons (across all axons diameters) from each animal that were of a specific diameter, which was fitted with a best-fit polynomial regression.

### Oligodendrocyte lineage cell cultures

Cortical mixed glial cultures generated from both male and female Sprague–Dawley rat P0–P2 pups were expanded for 10–14 days in vitro, and microglia depleted by de-adhesion following 1 h on a rotary shaker at 37 °C at 250 rpm. OPCs were subsequently isolated from collection of the floating fraction following 16 h on the rotary shaker, followed by depletion of contaminating astrocytes by differential adhesion. OPCs were plated in DMEM containing 4.5 g l^−1^ glucose, l-glutamine, pyruvate, SATO [16 µg ml^−1^ putrescine, 400 ng ml^−1^ l-thyroxine, 400 ng ml^−1^ tri-iodothyroxine, 60 ng ml^−1^ progesterone, 5 ng ml^−1^ sodium selenite, 100 µg ml^−1^ bovine serum albumin fraction V, 10 µg ml^−1^ insulin, 5.5 µg ml^−1^ halo-transferrin (all from Sigma-Aldrich)], 0.5% fetal calf serum (GIBCO), 1% penicillin/streptomycin, 10 ng ml^−1^ platelet-derived growth factor, and 10 ng ml^−1^ fibroblast growth factor-2, at 2 × 10^4^ cells per well in 8-well PDL-coated permanox chamberslides (Lab-TEK). OPCs were treated with activin-A (10 ng ml^−1^, R&D Systems) or vehicle control (0.0002% BSA) for 3 days. In a subset of experiments, OPCs were co-treated with activin-A and neutralizing antibodies against Acvr2a or Acvr2b (30 μg ml^−1^, R&D Systems; AF340, AF339) or goat IgG isotype control (30 μg ml^−1^, Santa Cruz Biotechnology). Cells were matured to oligodendrocytes by withdrawal of growth factors from the media and treated with activin-A or vehicle control, with or without neutralizing antibodies for 5 days as above. An average of 120 cells were counted per image, with 2 images assessed per condition per biological replicate (a total of > 700 cells quantified per condition); values were averaged per biological sample. For quantification of mature oligodendrocyte membrane area, images were imported into Columbus software (Perkin Elmer), individual cells identified (by the software) by Hoechst, oligodendrocytes identified by MBP staining, and size of cytoplasm measured in square pixels (px^2^) with a threshold of 2000 px^2^ set to exclude background/false positives. For Phalloidin intensity measurements, Columbus was used to first identify cells (Hoechst+ nuclei) then mature oligodendrocytes (MBP+), and Phalloidin signal intensity was measured in each MBP+ cell.

### Immunocytochemistry

Cells were fixed with 4% PFA (wt/vol) for 10–15 min and blocked for 30 min in 10% goat serum (Sigma), 2% horse serum (GIBCO), and 0.3% Triton-X-100 at 20–25 °C. Primary antibodies were diluted in blocking solution and applied for 1 h at 20–25 °C, and included mouse anti-MAG (1:100, Millipore; MAB1567), rat anti-MBP (1:250; AbD Serotec; MCA409S), and chicken anti-GFP (1:100; Abcam; ab13970). Cells were incubated with fluorescently conjugated secondary antibodies diluted in blocking solution (1:1000, Life Technologies-Molecular Probes), and in a subset of experiments with Phalloidin-Alexa-568 (1:40; ThermoFisher), for 1 h at 20–25 °C. Slides were counterstained with Hoechst (5 µg ml^−1^) and coverslipped with Fluoromount-G. Cells were imaged on a Leica SPE confocal microscope (40× objective) or an Olympus 3i Spinning Disk microscope (30× or 60× objectives).

### Lentiviral-based overexpression

Lentivirus particles generated using 3rd generation lentivirus packaging were purchased from Insight Bio/Origene (stock 10^7^ TU ml^−1^), inducing expression of GFP only for control transfection (PS100071V), or inducing overexpression of mouse Acvr2b and GFP (MR212153L2V). Particles were added to cultures of OPCs or differentiating oligodendrocytes at a multiplicity of infection (MOI) of 2.5 for 3 or 5 days, respectively. Cells were then fixed and immunostained or lysed for protein as above.

### Microfiber myelination assays

OPCs were plated onto PDL-coated parallel-aligned 2 μm electrospun poly-l-lactide acid (PLLA) fibers in 12-well plate inserts (The Electrospinning Company) at 35,000 cells per insert, as done previously [7]. Media was composed of 50:50 Neurobasal media and DMEM with high glucose supplemented with SATO [16 µg ml^−1^ putrescine, 400 ng ml^−1^ l-thyroxine, 400 ng ml^−1^ tri-iodothyroxine, 60 ng ml^−1^ progesterone, 5 ng ml^−1^ sodium selenite, 100 µg ml^−1^ bovine serum albumin fraction V, 10 µg ml^−1^ insulin, 5.5 µg ml^−1^ halo-transferrin), 5 µg ml^−1^ *N*-acetyl cysteine, 10 ng ml^−1^ d-Biotin (all from Sigma-Aldrich)], ITS supplement (Sigma), B27 (Invitrogen), and 1% penicillin/streptomycin. Cultures were treated with activin-A (1–100 ng ml^−1^, R&D Systems) or vehicle control for 14 days, fixed with 4% paraformaldehyde (wt/vol, Sigma), permeabilized with 0.1% Triton-X-100 (Fisher Scientific) and incubated overnight at 4 °C with rat anti-MBP antibody (1:250, AbD Serotec; MCA409S). Following three washes in PBS, inserts were incubated with secondary antibody (1:1000, Life Technologies-Molecular Probes) for 1 h at 20–25 °C, counterstained with Hoechst and mounted onto glass coverslips with Fluoromount G. Inserts were imaged as Z-stacks on an Olympus 3i Spinning Disk microscope (60×). Myelin sheath number per oligodendrocyte and lengths from 20 oligodendrocytes per biological preparation were analyzed in a blinded manner using the segmented line tool in Fiji/ImageJ (Fiji.sc) on maximum intensity images, using Z stacks to confirm complete wrapping around fibers.

### Forward phase phospho-antibody microarray

OPCs were plated at 1 × 10^6^ cells per well in a 6-well PDL-coated plate and treated with activin-A (10 ng ml^−1^) or vehicle control (0.0002% BSA). Cultures were washed with cold phosphate buffered saline on ice, scraped, and centrifuged thrice at 10,000 rpm at 4 °C and supernatant discarded. Protein extraction, lysate purification, and protein biotinylation were performed using the Antibody Array Assay Kit (Full Moon Biosystems) according to the manufacturer’s instructions. Samples were applied to TGF-β phospho-antibody microarray slides (Full Moon Biosystems) which have 176 immobilized antibodies against phosphorylated and unphosphorylated specific residues in proteins associated with the 5 TGFβ signaling pathways, with 6 technical replicates per antibody. All antibodies are against activating phosphorylation states, to the exception of Abl Thr754/735, GEF2 Ser885, Cofilin Ser3, Myc Ser373/Ser62/Thr358/Thr58. Following incubation with streptavidin-Cy3 (1:1000), signal was detected on an Axon4200 microarray scanner (Edinburgh Genomics, The University of Edinburgh). Subsequent to background signal subtraction, values from phosphorylated signal were normalized to total protein signal for each protein site, then normalized to vehicle control. Data were then Log2 transformed and plotted as heat maps using GraphPad Prism 7 (San Diego, USA).

### In vivo focal demyelinated lesions

Demyelinating lesions were induced in the caudal cerebellar peduncles of 12-week-old female Sprague–Dawley rats by stereotaxic injection of 4 µl of 0.01% ethidium bromide (vol/vol) using a Hamilton syringe. Rats were intracardially perfused with 4% PFA, cryoprotected, cryosectioned, and stained as above. Non-lesioned CCP served as a control.

### Post-mortem brain tissue

Formalin-fixed paraffin-embedded post-mortem perinatal brain injury tissue was obtained with full ethical approval from the Medical Research Council Edinburgh Brain and Tissue Bank (EBTB) (REC/1-/S1402/69). Tissue with evidence of white matter injury was selected with neuropathological support from Dr. Julie-Clare Becher (Royal Infirmary of Edinburgh) and Dr. Colin Smith (University of Edinburgh) (Table S1, Online Resource Supplemental Fig. 9a–c). Sections were de-paraffinized twice at 20–25 °C in Histoclear for 10 min then rehydrated through an ethanol gradient (100% (twice), 95, 70, and 50%, 5 min each). Following washes in 0.1% Tween20 (vol/vol) in Tris-buffered Saline (TBS), sections were microwaved in Vector Unmasking Solution for 10 min, washed once, and endogenous phosphatase and peroxidase activity blocked for 5 min (Bloxall, Vector). Primary antibody was prepared in 2.5% Normal Horse Serum (Vector) and applied overnight in a humid chamber at 4 °C. Antibodies used included mouse CD68 (1:100, DAKO; M0814), rat anti-MBP (AbD Serotec, 1:250; MCA409S), rabbit anti-INHBA (activin-A subunit; 1:100, Sigma-Aldrich; HPA020031), rabbit anti-Acvr2a (1:100, Abcam; ab135634), rabbit anti-Acvr2b (1:100, Abgent; AP7105a), rabbit anti-PCNA (1:400, Abcam; ab18197), and mouse anti-Olig2 (1:100, EMD Millipore; MABN50). Following washes, alkaline phosphatase-conjugated anti-mouse or anti-rabbit secondary antibody was applied for 30 min at 20–25 °C in a humid chamber. Sections were washed in TBS and stains visualized by Vector Blue substrate kit according to the manufacturer’s instructions (maximum 15 min). For co-staining, sections were washed thrice and re-blocked to quench any remaining phosphatase activity (Bloxall, Vector) prior to application of primary antibody, then developed using Vector Red substrate kit according to the manufacturer’s instructions (maximum 15 min). Following washes in water, the sections were counterstained with Hoechst and mounted with Fluoromount-G. Entire tissue sections were imaged using a Zeiss AxioScan SlideScanner. Due to non-neurological age-matched controls not being available, areas of injury (high CD68 densities; average 122 ± 30 cells/mm^2^) were compared to areas of non-injury (low CD68 densities; average 10 ± 10 cells/mm^2^) within the same section (pathology assessment shown in Online Resource Supplemental Fig. 9a–c). Lesion maps were defined for analysis in Zeiss Zen2 software; fields of 360 μm × 360 μm were counted per sample (injured or non-injured) and counts were multiplied to determine density of immunopositive cells per mm^2^. Post-mortem tissue from MS patients and controls who died of non-neurological causes were obtained via a UK prospective donor scheme with full ethical approval from the UK Multiple Sclerosis Tissue Bank (MREC/02/2/39). MS diagnosis was confirmed by neuropathological means by F. Roncaroli (Imperial College London) and clinical history was provided by R. Nicholas (Imperial College London). Snap frozen unfixed tissues blocks (2 × 2 × 1 cm) were cut at 10 µm and stored at − 80 °C. MS lesions were classified according to the International Classification of Neurological Disease using Luxol Fast Blue staining and Oil Red O immunoreactivity using standard methods (pathological assessment shown in Online Resource Supplemental Fig. 9d–f). We analyzed 4 control blocks and 10 tissue blocks from 8 MS patients (Table S2). Sections were fixed in 4% PFA for 1 h at 20–25 °C, washed in TBS and permeabilized in methanol for 10 min at − 20 °C. Sections were subsequently stained, imaged and quantified as above.

### Statistics

All manual cell counts were performed in a blinded manner. Data are represented as mean ± s.e.m. Power calculations for sample size were performed (using OpenEpi), and showed power between 84 and 100% for all experiments. Statistical tests include one-sample *t* test for data where values were normalized to control, two-tailed Student’s *t* test or Mann–Whitney test, and non-parametric one-way ANOVA (Kruskal–Wallis) with Dunn’s multiple comparison post hoc test when > 3 comparisons were made. A paired Student’s *t* test was used when variation between experiments was high, to normalize to baseline levels within each biological preparation. Slopes of myelin thickness versus axon diameter were compared using the Extra Sum of Squares F test. Curve distributions of proportion of myelinated axons per axon diameter were compared using the Kolmogorov–Smirnov test. Proportions of CC1+ or CC1− negative cells within the Olig2+ population were compared using Multiple *t* tests and two stage step up linear procedure of Benjamini, Krieger, and Yekutieli with a false discovery rate of 1%. *P* < 0.05 was considered to be statistically significant. Data handling and statistical processing were performed using Microsoft Excel and GraphPad Prism Software.

## Results

### Activin receptor signaling is required for developmental myelination

**Fig. 1:**
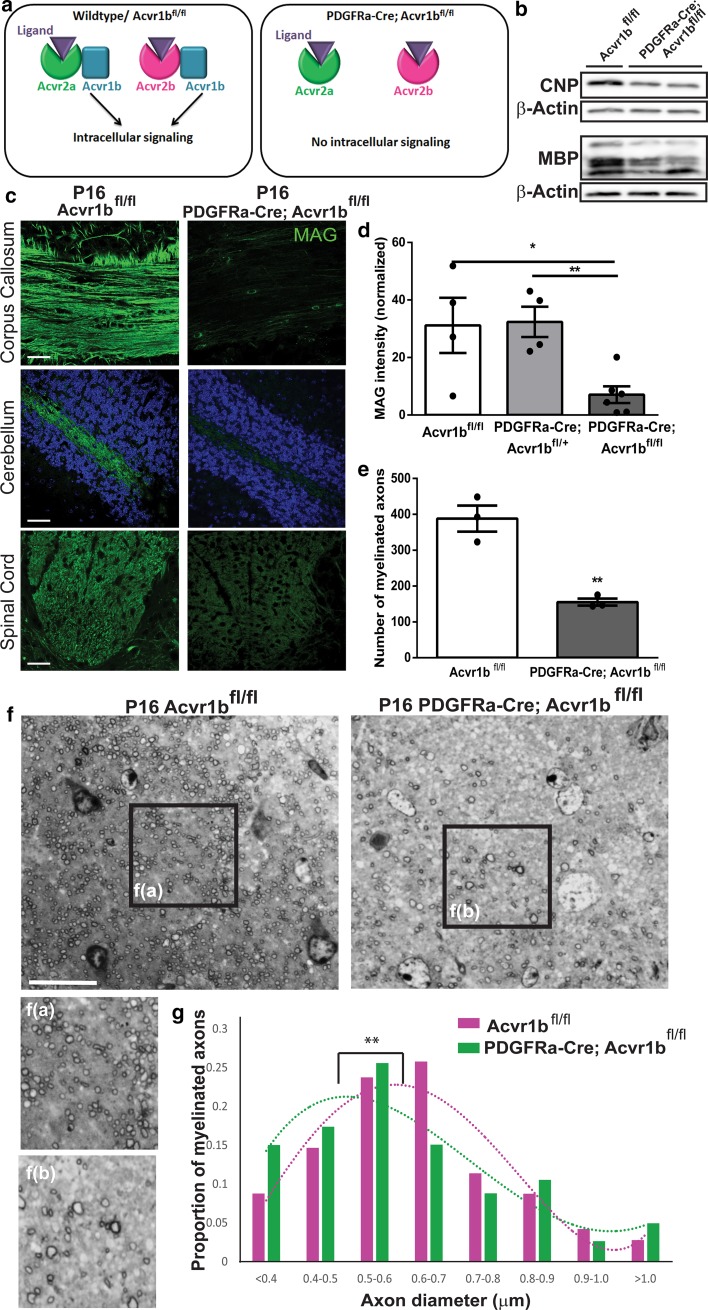
Activin receptor signaling is required for developmental myelination. **a** Diagram of activin receptor signaling: Acvr2a and Acvr2b are ligand-binding receptors that require Acvr1b to induce intracellular signallng. In PDGFRa-Cre; Acvr1b^fl/fl^ mice, the knockout of Acvr1b eliminates all activin receptor signaling from both ligand binding receptors. **b** Western blots of brain lysates from P16 Acvr1b^fl/fl^ and PDGFRa-Cre; Acvr1b^fl/fl^ mice (cerebellum) labeled for CNP (46 kDa) or MBP (14–21 kDa) with β-Actin as a loading control. **c** Images of corpus callosum, cerebellum [counterstained with Hoechst (blue)] and dorsal spinal cord in P16 Acvr1b^fl/fl^ and PDGFRa-Cre; Acvr1b^fl/fl^ mice immunostained for myelin protein MAG (green). Scale bar 25 μm. **d** Mean MAG intensity normalized to background ± s.e.m. in the corpus callosum of P16 Acvr1b^fl/fl^ (*n* = 4 mice), PDGFRa-Cre; Acvr1b^fl/+^ (*n* = 4 mice) and PDGFRa-Cre; Acvr1b^fl/fl^ mice (*n* = 6 mice). Two-tailed Student’s *t* test, *P = 0.0211, **0.0018. **e** Mean number of myelinated fibers ± s.e.m. per field of toluidine-blue stained semi-thin resin sections of corpus callosum at P16 in Acvr1b^fl/fl^ and PDGFRa-Cre; Acvr1b^fl/fl^ mice (*n* = 3 mice per group). Two-tailed Student’s *t* test, **P = 0.0034. **f** Toluidine-blue stained semi-thin resin sections of corpus callosum in Acvr1b^fl/fl^ (left) and PDGFRa-Cre; Acvr1b^fl/fl^ mice (right), with expanded field of view (**f**(a) and **f**(b), respectively). Scale bar 20 μm. **g** Analysis of distribution of myelinated axons in relation to axon diameter, represented as proportion of myelinated axons only (from all diameters), in Acvr1b^fl/fl^ (magenta) and PDGFRa-Cre; Acvr1b^fl/fl^ mice (green) mice (*n* = 3 mice per genotype) overlaid with polynomial best-fit regression curves (*R*^2^ = 0.8897, 0.8344, respectively). Kolmogorov–Smirnov test, **P = 0.002

To determine whether activin receptor signaling is required for developmental myelination, we sought to constitutively eliminate all activin receptor signaling in oligodendrocyte lineage cells. We achieved this by targeting *Acvr1b*, the activin co-receptor that is required for all intracellular signal transduction downstream of activin-A binding to either activin receptor Acvr2a or Acvr2b [6] (Fig. 1a). We thus created a constitutive conditional knockout in which oligodendroglial lineage cells cannot respond to any activin receptor ligation due to *Acvr1b* excision in OPCs (PDGFRa-Cre; Acvr1b^fl/fl^) [6, 53] (Fig. 1a; Online Resource Supplemental Fig. 1a–c). By immunofluorescence, Cre expression was confirmed to be in Olig2+ oligodendrocyte lineage cells (Online Resource Supplemental Fig. 1d). At postnatal day 16 (P16) when myelination is normally underway, PDGFRa-Cre; Acvr1b^fl/fl^ mice displayed a tremor, hunched posture, and a stiff tail (Online Resource Supplemental Video 1), all behaviors associated with myelin abnormalities [17, 44] or damage (e.g. in experimental autoimmune encephalomyelitis) [12, 58].

To determine whether this reflected myelin pathology, we assessed myelin-associated protein expression in the brains of Acvr1b^fl/fl^ control and PDGFRa-Cre; Acvr1b^fl/fl^ mice by Western blot and observed a decrease in 2′,3′-cyclic nucleotide 3′-phosphodiesterase (CNP) and myelin basic protein (MBP) in the conditional knockout mice (Fig. 1b). We further analyzed expression of myelin protein by immunofluorescence in multiple white matter tracts (corpus callosum, cerebellum, spinal cord) in PDGFRa-Cre; Acvr1b^fl/fl^ mice and observed a severe reduction in myelin-associated glycoprotein (MAG) intensity compared to Acvr1b^fl/fl^ control and heterozygous (PDGFRa-Cre; Acvr1b^fl/+^) littermates (Fig. 1c, d; Online Resource Supplemental Fig. 2a, b). Hypomyelination in PDGFRa-Cre; Acvr1b^fl/fl^ mice was further verified by histological analysis of semi-thin resin sections, which showed a significantly reduced density of myelinated axons by 60% (Fig. 1e, f). Peripheral nervous system myelin on dorsal root ganglia was unaffected in conditional knockout mice (Online Resource Supplemental Fig. 2c, d), as expected given the absence of PDGFRα expression in that compartment. Ultrastructural assessment of the sparse myelin that was produced by P16 in PDGFRa-Cre; Acvr1b^fl/fl^ mice revealed that of all the myelinated axons, the largest proportion was of a diameter of 0.5–0.6 μm, in comparison to that being 0.6–0.7 μm in floxed controls (Fig. 1g). A polynomial best-fit regression of the proportion of myelinated axons per axon diameter confirmed a statistically significant shift towards myelination of smaller diameter axons in activin receptor conditional knockouts (Fig. 1g). All PDGFRa-Cre; Acvr1b^fl/fl^ mice died by P21 precluding longitudinal analysis of myelination; juvenile death is also a feature of some other hypomyelinating mutants [9, 15, 18, 34, 54, 64]. Nonetheless, analysis of mice at P21 showed some myelination, although hypomyelination was still prevalent in the corpus callosum and cerebellum (Online Resource Supplemental Fig. 2e). Overall, these data show the requirement for activin receptor signaling in timely myelination of a significant proportion of CNS axons in development.

### Activin receptor signaling drives oligodendrocyte differentiation

**Fig. 2:**
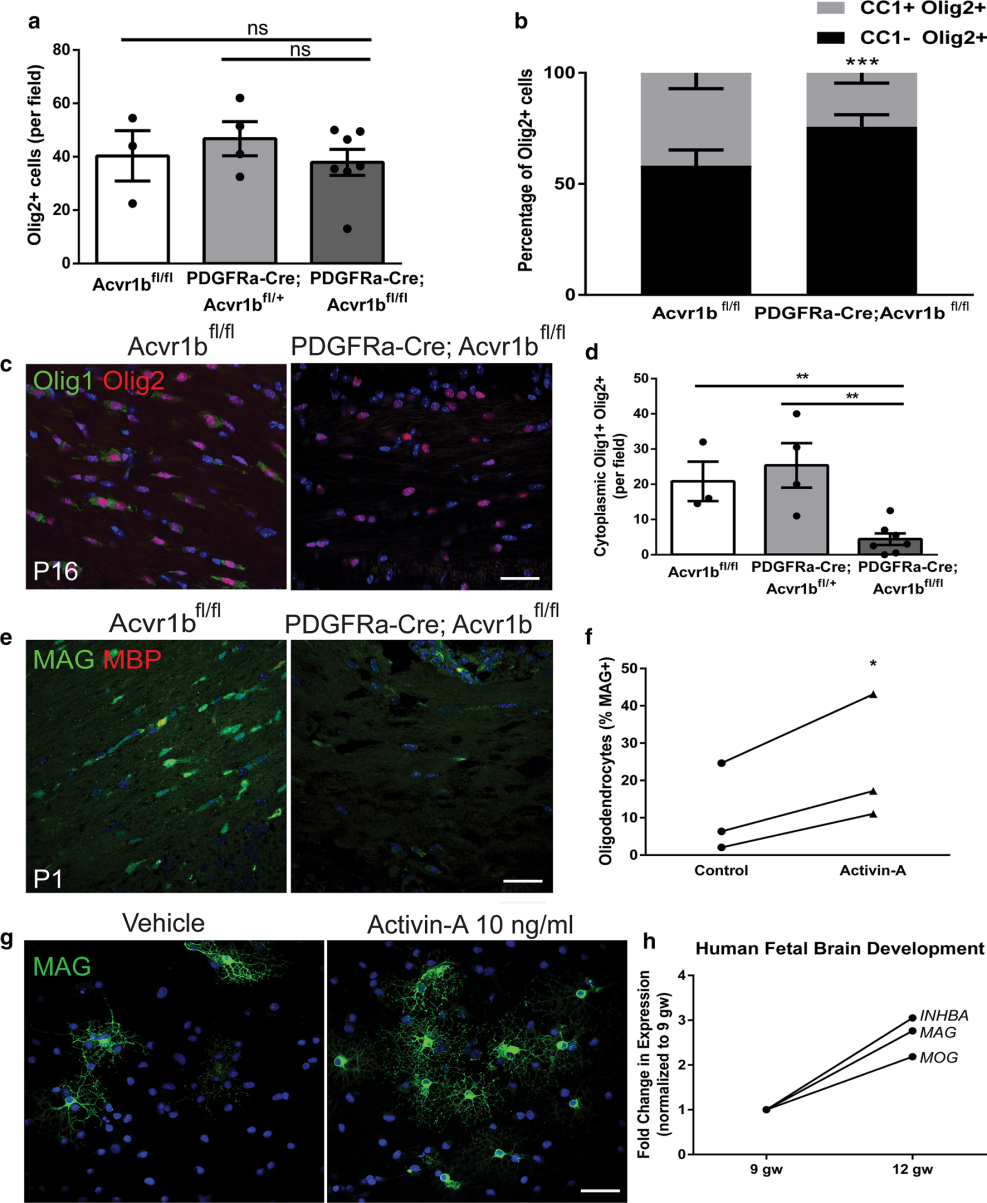
Activin receptor signaling regulates oligodendrocyte differentiation. **a** Mean number of oligodendrocyte lineage cells (Olig2+) per field ± s.e.m. in corpus callosum of P16 Acvr1b^fl/fl^ (*n* = 3 mice), PDGFRa-Cre; Acvr1b^fl/+^ (*n* = 4 mice) and PDGFRa-Cre; Acvr1b^fl/fl^ mice (*n* = 7 mice). **b** Mean proportion of oligodendrocyte lineage cells (Olig2+) which are mature oligodendrocytes (CC1+) versus immature cells (CC1−), per field ± s.e.m. in corpus callosum of P16 Acvr1b^fl/fl^ (*n* = 4 mice) and PDGFRa-Cre; Acvr1b^fl/fl^ mice (*n* = 7 mice). Multiple *t* tests with false discovery rate of 1%, ***P = 0.000026. **c** Images of differentiating oligodendrocytes (cytoplasmic Olig1+ and nuclear Olig2+) in corpus callosum of P16 Acvr1b^fl/fl^ and PDGFRa-Cre; Acvr1b^fl/fl^ mice. Scale bar 25 μm. **d** Mean number of cytoplasmic Olig1 and Olig2 double positive cells per field ± s.e.m. in corpus callosum of P16 Acvr1b^fl/fl^ (*n* = 3 mice), PDGFRa-Cre; Acvr1b^fl/+^ (*n* = 4 mice) and PDGFRa-Cre; Acvr1b^fl/fl^ mice (*n* = 7 mice). Two-tailed Student’s *t* test, **P = 0.0047, 0.0026, respectively. **e** Images of maturing oligodendrocytes (MAG+ MBP−) at P1 in corpus callosum of Acvr1b^fl/fl^ and PDGFRa-Cre; Acvr1b^fl/fl^ mice. Scale bar 25 μm. **f** Mean number of MAG+ cultured oligodendrocytes per field in vehicle control-treated or activin-A-treated conditions (10 ng ml^−1^) in vitro. *n* = 3 biological replicates. Two-tailed paired Student’s *t* test, *P = 0.0484. **g** Images of cultured OPCs treated with vehicle or 10 ng ml^−1^ activin-A and immunostained for MAG (green), counterstained with Hoechst (blue). Scale bar 25 μm. **h** Data-mining of microarray of human fetal brain at 9 and 12 gestational weeks (gw) represented as fold change in expression (normalized to 9 gw), showing paralleled expression changes between activin-A (*INHBA*) and oligodendrocyte differentiation-associated genes (*MAG*, *MOG*) in development

We next sought to determine the cellular mechanisms underpinning the hypomyelination in PDGFRa-Cre; Acvr1b^fl/fl^ mice, by assessing oligodendroglial responses. Between genotypes at P16, there were no differences in total oligodendroglial lineage cell number (Olig2+) (Fig. 2a); we did not observe cell death in the lineage at either P16 or earlier (cleaved caspase-3 negative and TUNEL negative) in any genotype (Online Resource Supplemental Fig. 3), consistent with oligodendrocyte death normally occurring later in development [24]. However, whereas in floxed control mice, the proportion of Olig2+ cells that were mature oligodendrocytes (CC1+) was not significantly different from that which were immature cells (CC1−), PDGFRa-Cre; Acvr1b^fl/fl^ mice had significantly less CC1+ Olig2+ cells versus CC1− Olig2+ cells (Fig. 2b), suggesting impaired differentiation into mature oligodendrocytes. To specifically address this, we quantified differentiating oligodendrocytes (cytoplasmic Olig1+ Olig2+ cells) and found that these were significantly decreased in PDGFRa-Cre; Acvr1b^fl/fl^ mice compared to controls (Fig. 2c, d). The oligodendrocyte maturation defect in activin receptor conditional knockout mice was observed as early as P1, when few immature oligodendrocytes (MAG+ MBP−) cells were observed (Fig. 2e). These data support that activin receptor signaling is required for differentiation of a significant proportion of oligodendrocyte lineage cells.

To assess the sufficiency of activin receptor signaling in driving oligodendrocyte differentiation, primary cultures of wildtype OPCs were treated with the most potent activating ligand, activin-A. Even in the presence of proliferation-inducing growth factors (platelet-derived growth factor and fibroblast growth factor) which normally preclude differentiation, activin-A significantly enhanced OPC differentiation into mature oligodendrocytes (MAG+) (Fig. 2f, g). Consistent with this finding, data-mining of the human fetal brain transcriptome [39] revealed that as development proceeds, increased gene expression of activin-A (*INHBA*) parallels that of oligodendrocyte maturation-associated genes *MAG* and *MOG* (Fig. 2h). The activin-A subunit (Inhba) was also found to be expressed in developing mouse brain during myelination (Online Resource Supplemental Fig. 4).

### Activin receptor signaling drives oligodendrocyte membrane compaction

**Fig. 3:**
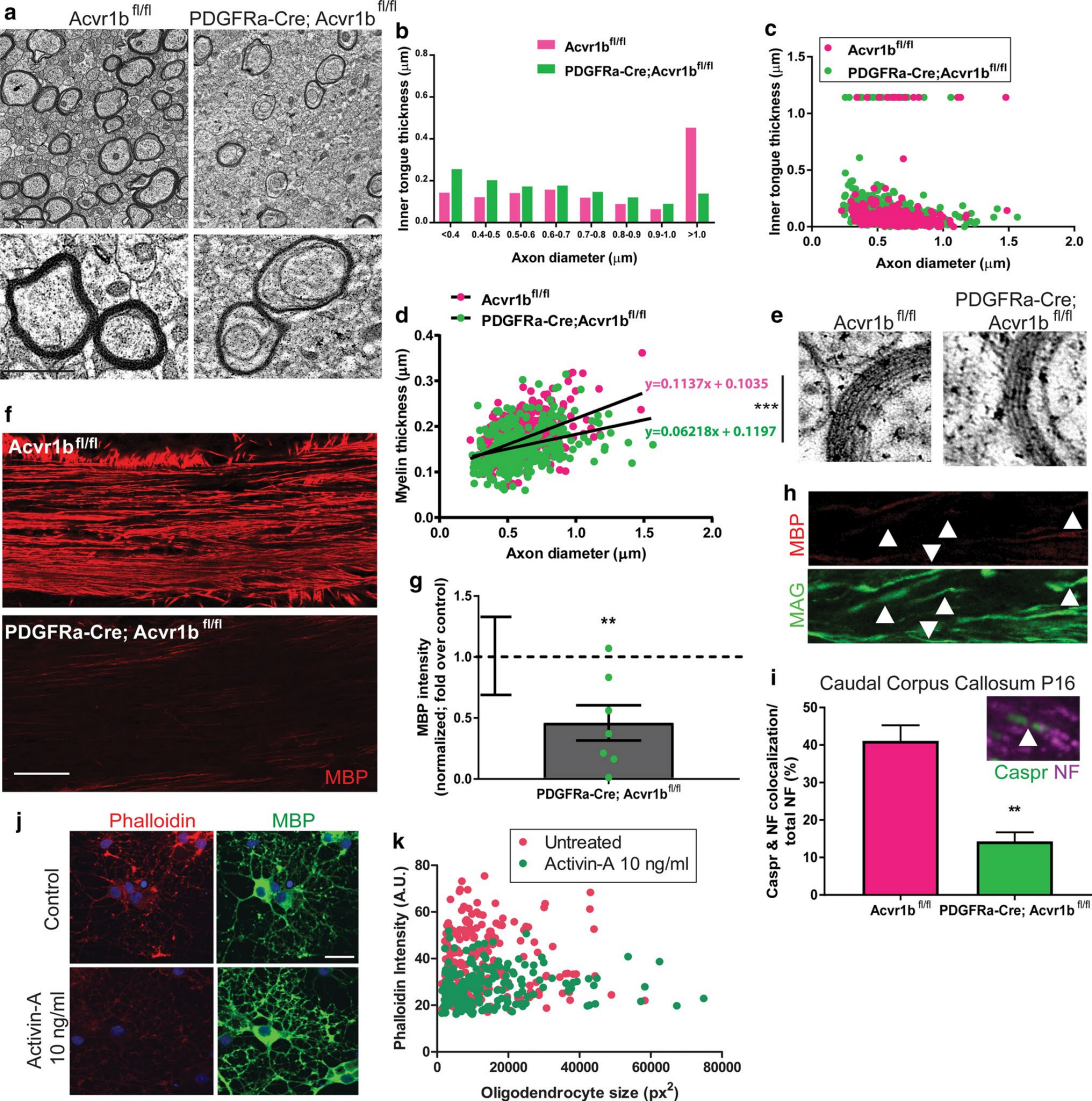
Activin receptor signaling regulates myelin membrane compaction/maturation. **a** Electron micrographs of myelinated axons in P16 Acvr1b^fl/fl^ and PDGFRa-Cre; Acvr1b^fl/fl^ mice. Scale bars 1 μm. **b** Average inner tongue thickness per axon diameter per animal in Acvr1b^fl/fl^ (magenta) and PDGFRa-Cre; Acvr1b^fl/fl^ mice (green). *n* = 3 mice per genotype. **c** Dot plot of inner tongue thickness per axon diameter for all myelinated axons for all animals, in Acvr1b^fl/fl^ (magenta) and PDGFRa-Cre; Acvr1b^fl/fl^ mice (green). **d** Myelin thickness versus axon diameter in Acvr1b^fl/fl^ (magenta) and PDGFRa-Cre; Acvr1b^fl/fl^ mice (green). Extra sum of squares F test between slopes, ***P = 0.0014. **e** Electron micrographs of compact myelin layers in P16 Acvr1b^fl/fl^ and PDGFRa-Cre; Acvr1b^fl/fl^ mice. **f** Images of myelin basic protein (MBP) in corpus callosum of Acvr1b^fl/fl^ and PDGFRa-Cre; Acvr1b^fl/fl^ mice at P16. Scale bar 25 μm. **g** MBP intensity in PDGFRa-Cre; Acvr1b^fl/fl^ mice normalized to background and to levels in Acvr1b^fl/fl^ mice. s.e.m. for variance in Acvr1b^fl/fl^ samples indicated. One sample *t* test against theoretical mean of 1, **P = 0.0097. **h** Image of PDGFRa-Cre; Acvr1b^fl/fl^ corpus callosum showing MAG+ (green), MBP negative (red) myelin sheaths (arrows). **i** Percentage of total axonal area (neurofilament (NF)+) co-localizing with compaction marker Caspr in caudal corpus callosum at P16 in Acvr1b^fl/fl^ and PDGFRa-Cre; Acvr1b^fl/fl^ mice. Two-tailed Student’s *t* test, **P = 0.0038, *n* = 2–4 mice per group. Inset example of Caspr clusters (green) at paranodes along axon (NF+; purple) (arrow). **j** Images of cultured mature oligodendrocytes treated with vehicle control or activin-A (10 ng ml^−1^) stained with Phalloidin-Alexa-568 (red) and MBP (green). Scale bar 20 µm. **k** Phalloidin intensity (arbitrary units; A.U.) in MBP+ sheets of mature oligodendrocytes plotted against oligodendrocyte size (pixels squared; px^2^) in control (magenta) or activin-A (10 ng ml^−1^) treated (green) conditions

Although we demonstrated impaired oligodendrocyte differentiation in the activin receptor conditional mutant mouse, there were still some oligodendrocytes that were generated resulting in limited myelination taking place. We next analyzed whether this myelin was normally formed, to determine whether activin receptor signaling is required for proper myelination. In PDGFRa-Cre; Acvr1b^fl/fl^ mice at P16, we observed increased myelin inner tongue thickness in PDGFRa-Cre; Acvr1b^fl/fl^ mice compared to floxed controls (Fig. 3a–c), most prominently in association with small-diameter axons (Fig. 3b, c; measurement protocol outlined in Online Supplemental Resource Fig. 5). Enlarged myelin inner tongues are normally seen in early myelination during the active growth phase, resulting from accumulation of new myelin membrane prior to actin disassembly mediated compaction and fusion with myelin sheaths [48, 60, 66]. Persistent enlargement of inner tongues may thus result either from (i) increased membrane growth rate, or (ii) impaired actin disassembly and myelin membrane compaction [48, 61, 66]. Given that the former postulate would result in a thicker myelin sheath, we measured thickness of compacted layers (as done previously [66]; measurement protocol outlined in Online Supplemental Resource Fig. 5), yet documented significantly thinner myelin at all axon diameters in PDGFRa-Cre; Acvr1b^fl/fl^ mice (Fig. 3d) thereby ruling out increased membrane growth. The thinner myelin in conditional knockout mice was confirmed to be compact (Fig. 3e). To address the latter postulate, we assessed expression of myelin basic protein (MBP), which is required for actin disassembly and myelin membrane compaction [60, 66]. PDGFRa-Cre; Acvr1b^fl/fl^ mice showed significantly decreased MBP intensity relative to floxed controls (Fig. 3f, g); MAG+ myelin sheaths devoid of MBP were also observed in these mice (Fig. 3h), indicative of non-compact myelin as MAG is normally excluded from compact myelin sheaths by MBP [2, 3]. These data suggest that activin receptor signaling in oligodendroglial lineage cells is required for normal MBP expression and myelin membrane compaction.

We next asked whether this impairment in compaction would be maintained as myelination proceeds in the conditional knockout mice. Due to these mice dying by P21, we addressed this by examining the caudal region of the corpus callosum in P16 mice, where myelination is more advanced relative to rostral regions. As clustering of the axonal adhesion molecule Caspr along axons only occurs when myelin is compacted at the internode [63], we used the percentage of axonal area co-localizing with Caspr clusters as a readout of compaction. In comparison to Acvr1b^fl/fl^ mice, the percentage of total axonal area (NF+) occupied by Caspr clusters was significantly lower in caudal corpus callosum of PDGFRa-Cre; Acvr1b^fl/fl^ mice, indicative of a maintained deficiency in myelin compaction (Fig. 3i).

Given that myelin compaction requires actin disassembly [48, 66], the sufficiency of activin receptor signaling in accelerating actin depolymerization in oligodendrocyte membranes was assessed. Maturing wildtype oligodendrocytes were treated with activin-A, and non-depolymerized filamentous actin was detected using fluorescently conjugated Phalloidin, a method established in recent studies of actin dynamic-driven oligodendrocyte membrane compaction [48, 66]. Oligodendrocyte membranes in control conditions had high Phalloidin signal throughout MBP+ membranes at 5 days of in vitro maturation, indicative of non-depolymerized actin (average intensity/cell 38.3 ± 0.9 A.U.; Fig. 3j, k). Conversely, activin-A-treated oligodendrocyte membranes showed a reduced Phalloidin intensity, demonstrating increased actin depolymerization consequent to activin receptor signaling (average intensity/cell of 28.0 ± 0.5 A.U.; Fig. 3j, k).

To next test whether activin signaling regulates the total amount of myelin membrane made by oligodendrocytes, we first measured the average size of oligodendrocyte membranes in activin-A-treated cultures, yet found no difference to vehicle-treated cultures (Fig. 3k and Online Resource Supplemental Fig. 6a, b). We assessed the number and length of myelin sheaths formed by oligodendrocytes on poly-L-lactic acid microfibers, and also found no significant effect of activin-A treatment (Online Resource Supplemental Fig. 6c, d). Overall these results demonstrate that although activin receptor signaling does not increase the amount of membrane produced once an oligodendrocyte has differentiated, it has an important role in efficient oligodendrocyte membrane actin disassembly and myelin compaction.

### Activin receptor subtype Acvr2a regulates oligodendrocyte differentiation and membrane actin disassembly

**Fig. 4:**
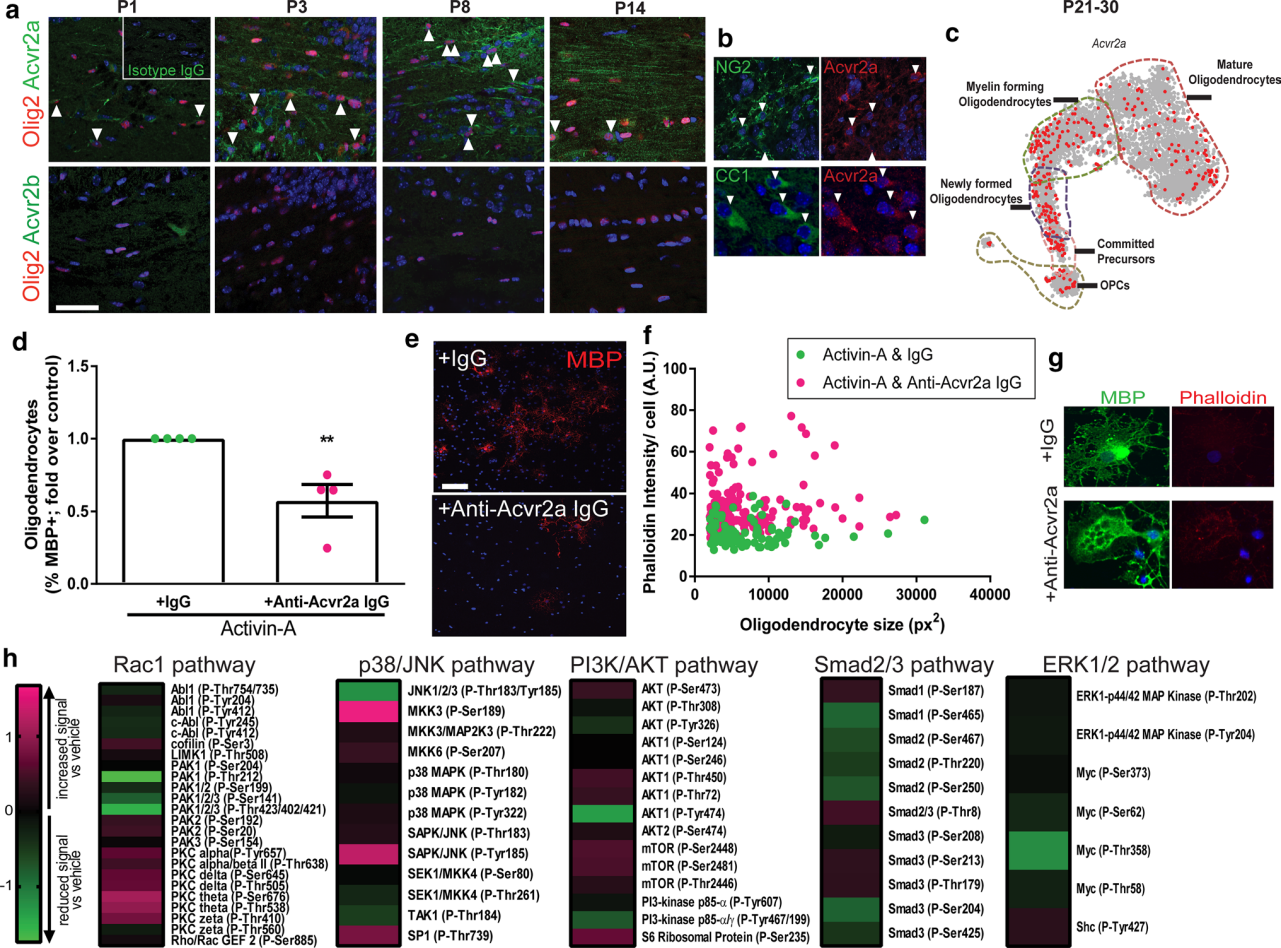
Activin receptor Acvr2a regulates oligodendrocyte lineage cell behavior. **a** Acvr2a (top row; green) and Acvr2b (bottom row; green) expression by oligodendrocyte lineage (Olig2+; red) throughout development (P1–P14; double positive cells indicated by arrows), counterstained with Hoechst (blue). Inset; isotype control for Acvrs. Scale bar 50 μm. **b** Expression of Acvr2a (red) by NG2+ cells (green) (top row), and by CC1+ cells (green) (bottom row). **c** Data-mining of oligodendrocyte lineage cell transcriptomes from P21–30 for *Acvr2a* expression, represented as *t* distributed stochastic neighbor embedding projection. **d** OPCs co-treated with activin-A and neutralizing antibodies for Acvr2a or isotype IgG. Mean percentage of oligodendrocytes (MBP+) normalized to isotype control ± s.e.m. *n* = 3 biological replicates, two-tailed Student’s *t* test, *P* = 0.0087. **e** Images of MBP+ cells (red) in cultures treated with activin-A (10 ng ml^−1^) and isotype IgG or Acvr2a-neutralizing IgG. Scale bar 75 μm. **f** Phalloidin intensity signal (arbitrary units; AU) per oligodendrocyte plotted against oligodendrocyte size (px^2^) in cultures co-treated with activin-A (10 ng ml^−1^) and IgG control or neutralizing antibody against Acvr2a. *n* = 3 biological replicates. **g** Images of MBP+ oligodendrocytes (green) and filamentous actin (Phalloidin+; red) in cultures treated with activin-A and IgG or Acvr2a neutralizing antibody. **h** Log2-transformed phosphorylation signal of TGFβ superfamily pathways following treatment with activin-A, normalized to respective total protein signal then to vehicle control. Heat map: compared to vehicle, magenta indicates increased signal, black no change, and green reduced signal

Having shown that signal transduction via the co-receptor Acvr1b is required for oligodendrocyte differentiation and membrane compaction, we next asked which ligand-binding activin receptor drives this effect. Activin-A can bind to two activin receptor subtypes, Acvr2a and Acvr2b; whether these have differential function or protein expression patterns in the CNS is unknown. We assessed Acvr2a and Acvr2b expression in the oligodendrocyte lineage during developmental myelination. Acvr2a expression in the cell bodies of Olig2+ cells was found to progressively increase over time while oligodendrocyte differentiation is underway (P1–P8), and was later also associated with myelin at the peak of myelination (P14) (Fig. 4a). We detected Acvr2a expression in both NG2+ immature cells and CC1+ mature oligodendrocytes (Fig. 4b). Data-mining of oligodendrocyte lineage single-cell transcriptomes in later development (P21–30; [41]; linnarssonlab.org/oligodendrocytes) confirmed sustained *Acvr2a* expression throughout the lineage, including in subsets of precursors and myelinating oligodendrocytes (Fig. 4c). Conversely, Acvr2b protein was not detectable at any time point in development (Fig. 4a), in contrast to the robust signal observed in positive control placental tissue (Online Resource Supplemental Fig. 7a) [56], and consistent with undetectable *Acvr2b* mRNA in the developing brain at the onset of myelination (Online Resource Supplemental Fig. 7b).

Given that Acvr2a is the receptor subtype expressed at the onset of differentiation and myelination, we next tested whether this receptor drives these responses by co-treating oligodendrocyte lineage cells with activin-A and an Acvr2a-specific neutralizing antibody, or an isotype IgG control. Protein blasting the epitope sequences for the Acvr2a antibody showed 100% specificity and selectivity for this receptor subtype. Blocking Acvr2a reversed the effects of activin-A on differentiation of OPCs into mature oligodendrocytes (MBP+) (Fig. 4d, e), and prevented activin-A-driven actin disassembly in oligodendrocytes (causing increased Phalloidin intensity in MBP+ membranes; Fig. 4f, g). Conversely, blocking Acvr2b had no significant effect on oligodendrocyte differentiation (Online Resource Supplemental Fig. 8a), although it did reduce activin-A-driven actin disassembly (Online Resource Supplemental Fig. 8b). Nevertheless, this data revealed that Acvr2a is the activin receptor subtype expressed by the oligodendrocyte lineage during developmental myelination and can drive activin-A-mediated oligodendrocyte differentiation and membrane actin disassembly.

To next determine which signaling pathways mediate Acvr2a-driven oligodendrocyte differentiation and membrane actin depolymerization, we assessed activation of the 5 TGFβ signaling pathways induced downstream of activin receptors (Rac1 GTPase, p38 MAPK/JNK, PI3K/AKT, Smad2/3, ERK1/2,) which are also associated with regulation of oligodendroglial lineage responses and myelination [4, 27, 29, 47, 61]. Activation of all pathways in activin-A-stimulated oligodendroglial lineage cells was simultaneously assessed by measuring phosphorylation levels of signaling proteins using a forward-phase antibody microarray; phosphorylation signal was normalized to respective total protein signal, and values then normalized to vehicle control. We found that activin-A increased phosphorylation signal in all pathways, with the highest increase in signal observed in the Rac1 GTPase and p38/MAPK pathways (Fig. 4h). Taken together with our findings, these results are consistent with known roles for the Rac1 GTPase and p38 MAPK pathways in regulating oligodendrocyte differentiation, myelination, and myelin compaction [11, 13, 19, 25, 29, 61].

### Activin receptor signaling regulates remyelination

**Fig. 5:**
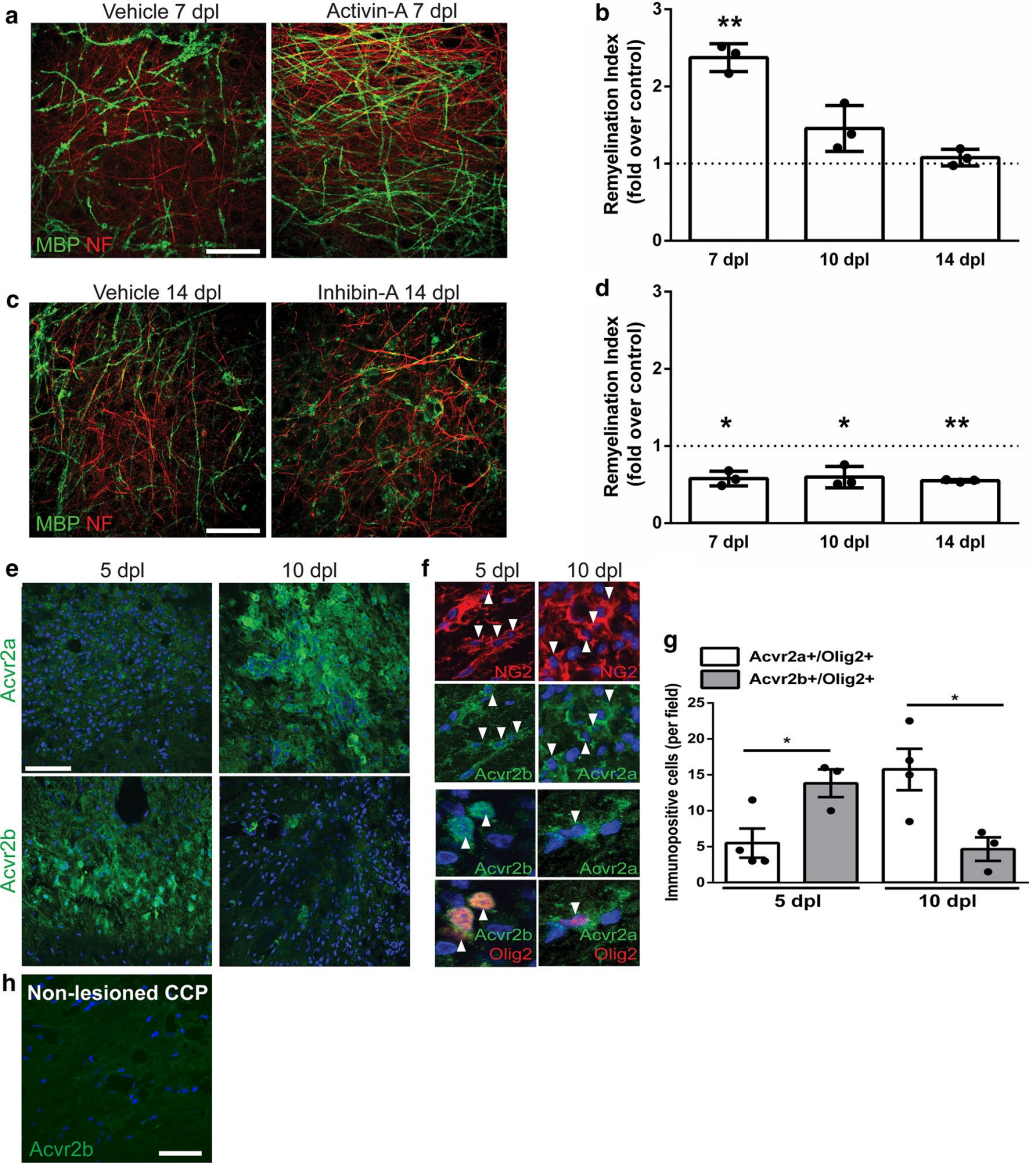
Activin receptor signaling regulates remyelination. **a** Representative images of organotypic cerebellar slice cultures at 7 days post lysolecithin-induced demyelination, treated with vehicle control or activin receptor agonist activin-A during remyelination, immunostained against myelin basic protein (MBP; green), and axonal neurofilament-H (NF; red). Scale bar 50 μm. **b** Mean remyelination index ± s.e.m. in activin-A-treated explants at 7, 10, and 14 days post lysolecithin (dpl) normalized to vehicle control from the respective time point. *n* = 3 animals, one-sample *t* test compared to theoretical mean of 1 (control), **P = 0.0057. **c** Representative images of slice cultures at 14 dpl treated with vehicle control or an inhibitor of activin receptor signaling inhibin-A during remyelination, immunostained against myelin basic protein (MBP; green) and axonal neurofilament-H (NF; red). Scale bar 50 μm. **d** Mean remyelination index ± s.e.m. in inhibin-A-treated explants at 7, 10, and 14 dpl normalized to vehicle control from the respective time point. *n* = 3 animals, one-sample *t* test compared to theoretical mean of 1 (control), *P = 0.0165, *0.0374, **0.0004, respectively. **e** Acvr2a and Acvr2b expression (green) in demyelinated caudal cerebellar peduncles (CCP) at 5 days post-lesion (dpl; prior to remyelination) and 10 dpl (onset of remyelination), counterstained with Hoechst (blue). Scale bar 25 μm. **f** Colocalization of Acvr2b or Acvr2a (green) with NG2 (top 2 rows; red; arrowheads) or Olig2 (bottom 2 rows; red; arrowheads) at 5 and 10 dpl in CCP, counterstained with Hoechst (blue). **g** Mean number of cells double positive for Olig2 and Acvr2a or Acvr2b per field ± s.e.m. at 5 and 10 dpl. *n* = 3 = 4 animals per group. Two-tailed Student’s *t* test, *P* = 0.0345 (5 dpl), 0.0298 (10 dpl). **h** Non-lesioned CCP shows no staining of Acvr2b (green). Scale bar 10 μm

Having shown the role of activin receptor signaling in developmental myelination, we next asked whether it is also relevant to regeneration of myelin following injury. Our previous work showed that at the onset of remyelination, activin-A levels increase and activin receptors are expressed by OPCs [45]. To examine whether this expression has functional significance for remyelination, we used activin-A to stimulate activin receptors during remyelination of ex vivo organotypic cerebellar brain explants which were demyelinated with lysolecithin. Activin-A significantly increased remyelination at 7 days post lysolecithin (dpl) compared to vehicle control, as measured by remyelination index (co-localization of myelin basic protein (MBP) and axonal neurofilament, normalized to area of neurofilament) [63, 65] (Fig. 5a, b). Due to remyelination subsequently occurring efficiently in controls, no significant differences between vehicle and activin-A-treated explants were observed at later time points (10, 14 dpl) (Fig. 5b). These data demonstrate that activin receptor stimulation is sufficient to accelerate remyelination. To determine whether activin receptor signaling is required for remyelination, demyelinated cerebellar explants were treated with an inhibitor of activin receptors, inhibin-A, which competitively binds these receptors to prevent endogenous ligand binding [42]. Remyelination was significantly repressed by inhibin-A treatment at 7, 10 and 14 dpl compared to vehicle control (Fig. 5c, d), demonstrating for the first time that activin receptor signaling is required for remyelination.

To assess activin receptor subtype expression during efficient remyelination, we took advantage of the temporally discrete OPC responses occurring following focal demyelinated lesion induction of the caudal cerebellar peduncles. At the time of initiation of oligodendrocyte differentiation and remyelination (10 dpl), Acvr2b levels decreased and Acvr2a levels concomitantly increased (Fig. 5e, g); these changes in expression were confirmed to occur in oligodendroglial lineage cells (NG2+, Olig2+; Fig. 5f). Non-lesioned CCP had no Acvr2b signal, confirming the absence of its expression in healthy adult white matter (Fig. 5h). Thus, in contrast to the absence of Acvr2b expression during development or homeostasis, its transient induction after demyelination is an early response to injury, which is followed by a transition to Acvr2a expression during efficient remyelination.

### Activin receptor expression is dysregulated in non-repairing lesions in human myelin disorders

**Fig. 6:**
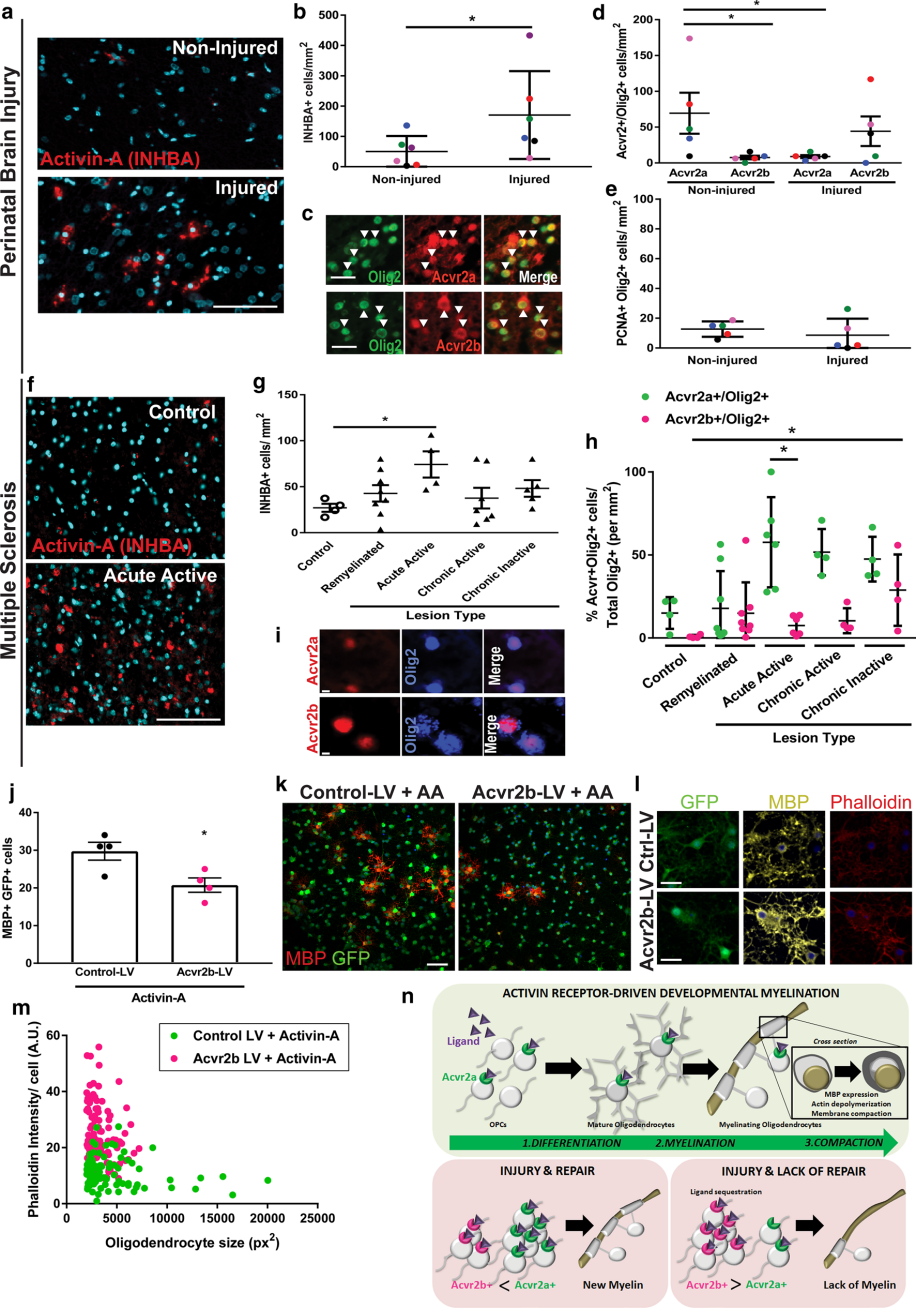
Activin receptor expression dysregulation in developmental and adult human myelin disorders. **a** Images of activin-A subunit (INHBA; red) immunostaining in non-injured and injured developing white matter in a case of perinatal brain injury, counterstained with Hoechst (turquoise). Scale bar 25 μm. **b** Mean densities of INHBA+ cells ± s.e.m. per mm^2^ in non-injured and injured developing white matter in perinatal brain injury. *n* = 5 cases (Table S1); each patient block is represented by different color. Mann–Whitney test, *P = 0.0411. **c** Images of oligodendrocyte lineage cells (Olig2+; green) expressing Acvr2a or Acvr2b (red) in developing white matter, indicated by arrowheads. Scale bar 25 μm. **d** Densities of Acvr2a+ Olig2+ and Acvr2b+ Olig2+ cells per mm^2^ in non-injured versus injured areas of developing white matter. *n* = 5 cases; each patient block is represented by different color. Mann–Whitney test, *P = 0.0238 (non-injured Acvr2a+ Olig2+ vs Acvr2b+ Olig2+), *P = 0.0317 (non-injured Acvr2a+ Olig2+ vs injured Acvr2a+ Olig2+). **e** Densities of PCNA+ Olig2+ proliferating oligodendrocyte lineage cells per mm^2^ in non-injured vs injured areas of developing white matter. *n* = 5 cases; each patient block represented by different color. **f** Images of INHBA+ cells (red) in control and acute active multiple sclerosis (MS) lesion tissue, counterstained with Hoechst (turquoise). Scale bar 100 μm. **g** Mean densities of INHBA+ cells per mm^2^ in post-mortem brain tissue from healthy control, or MS lesions (remyelinated, acute active, chronic active (rim), chronic inactive). *n* for each lesion type indicated in Table S2. Mann–Whitney test, *P = 0.0286. **h** Proportion of Acvr2a+ Olig2+ or Acvr2b+ Olig2+ from total Olig2+ cells in healthy control tissue or MS lesions (remyelinated, acute active, chronic active (rim), chronic inactive). *n* for each lesion type indicated in Table S2. Kruskal–Wallis test and Dunn’s multiple comparison test, *p < 0.05. **i** Acvr2a+ (top row; red) or Acvr2b+ (bottom row; red) and Olig2+ (blue) double positive cells in MS lesions. Scale bar 5 μm. **j** Quantification of differentiation of OPCs into mature oligodendrocytes (MBP+; per field) following transfection with lentivirus (GFP+), either control (control-LV) or Acvr2b-expressing (Acvr2b-LV), and treated with activin-A (10 ng ml^−1^) for 3 days. *P = 0.0247, two-tailed Student’s *t* test. **k** Representative images of OPC cultures treated with activin-A and Control-LV or Acvr2b-LV for 3 days immunostained for MBP (red) and GFP (green). Scale bar 50 µm. **l** Representative images of maturing oligodendrocytes transfected with Control-LV or Acv2b-LV for 3 days and treated with activin-A (10 ng ml^−1^) for 5 days, stained for GFP (green), MBP (false colored yellow), and Phalloidin (red). Scale bar 20 µm. **m** Phalloidin intensity signal (arbitrary units; AU) per MBP+ transfected (GFP+) oligodendrocyte plotted against oligodendrocyte size (px^2^) in cultures co-treated with activin-A (10 ng ml^−1^) and Control-LV or Acvr2b-LV. **n** Model for role of activin receptor signaling in oligodendrocyte lineage cells. Acvr2a is expressed during developmental myelination, inducing oligodendrocyte differentiation, myelination, and myelin membrane compaction. Following injury, successful repair involves a transition in expression from Acvr2b to Acvr2a to support new myelin formation. In myelin disorders, failed repair is associated with an upregulation of Acvr2b, impairing Acvr2a-driven responses, leading to lack of myein

In light of our results showing that activin receptor signaling regulates developmental myelination and remyelination, and that activin receptor subtype expression by oligodendroglial lineage cells is strictly controlled following injury, we predicted that dysregulation of activin receptor expression may underpin chronic failure of oligodendrocyte differentiation and myelin formation in human disorders [10, 38]. We first investigated brain tissue from perinatal brain injury cases (Online Resource Supplemental Table 1) and compared areas of normally developing white matter (low microglia density: average 10 ± 10 cells/mm^2^) to areas of injury (high microglia density: average 122 ± 30 cells/mm^2^) (Online Resource Supplemental Fig. 9a–c). We observed increased densities of cells positive for the activin-A subunit INHBA in the brain parenchyma in injured vs. non-injured areas (Fig. 6a, b), consistent with previously reported elevated INHBA in the cerebrospinal fluid following perinatal brain injury [23]. However, injured areas had lower densities of oligodendroglial cells (Olig2+) expressing Acvr2a, and higher densities of those expressing Acvr2b (Fig. 6d), compared to non-injured areas. This was not associated with a normal regenerative response to injury, as proliferating oligodendroglial lineage cells (PCNA+ Olig2+) were not increased relative to in non-injured regions (Fig. 6e).

We next examined adult progressive MS brain lesions with four types of pathology displaying a range of remyelination potential: lesions with completed remyelination (‘remyelinated’), ongoing damage and active remyelination (‘acute active’, rim of ‘chronic active’), and little to no remyelination (‘chronic inactive’) (Online Resource Supplemental Table 2, Online Resource Supplemental Fig. 9d–f). Activin-A (INHBA)+ cells were present in lesions regardless of pathology, yet densities were significantly increased in acute active lesions (Fig. 6f, g) consistent with increased INHBA detected in cerebrospinal fluid following recent adult brain injury [52]. Within actively remyelinating lesions (acute active and chronic active lesions), the proportion of Olig2+ cells that were Acvr2a+ was higher than the proportion that were Acvr2b+ (Fig. 6h). However, the proportion of Acvr2b+ Olig2+ cells was significantly increased in chronic inactive lesions (Fig. 6h), the lesions with the lowest remyelination potential. Thus, a common feature of lesions with limited oligodendrocyte differentiation and myelin generation in both developmental and adult disorders was an increase in Acvr2b+ oligodendroglial lineage cells. Both the increase in Acvr2b expression and its relatively higher affinity for activin-A [5] would be predicted to sequester the ligand thereby restricting Acvr2a-mediated signaling in oligodendrocyte lineage cells.

To test this hypothesis, Acvr2b was overexpressed in oligodendrocyte lineage cells via lentiviral delivery of a construct driving expression of Acvr2b along with a membrane-tagged green fluorescent protein (GFP) (Acvr2b-LV), and using a lentivirus driving GFP expression only as a control (control-LV). Successful transfection of OPCs at 3 days of exposure was confirmed by detection of GFP expression in 99% of cells (Fig. 6k). Compared to control-LV conditions, overexpression of Acvr2b significantly impaired both activin-A-driven OPC differentiation into mature MBP+ oligodendrocytes (Fig. 6j, k) and actin depolymerization in maturing oligodendrocyte membranes (causing an increase in Phalloidin intensity per cell; Fig. 6l, m). These experiments confirm that increased expression of Acvr2b by oligodendroglial lineage cells impairs ligand-driven oligodendrocyte differentiation and actin dynamics involved in myelin compaction.

## Discussion

In this study, we reveal the role of activin receptor signaling in CNS myelin generation during healthy development and following injury. Using a conditional knockout approach, in vitro manipulation of oligodendrocyte lineage cells, ex vivo/in vivo modeling of remyelination, and human post-mortem brain tissue analysis, we uncover the requirement for activin receptor signaling in driving timely oligodendrocyte differentiation and myelin compaction, and its dysregulation in disease (summarized in Fig. 6n). These findings go beyond previous studies showing that activin receptor stimulation is protective for neurons [22, 30, 55], by demonstrating a direct and disease-relevant role in oligodendrocyte lineage cells. Our results also extend the functions of the TGFβ superfamily beyond supporting oligodendrocyte differentiation [19, 49] to now include regulation of myelin compaction and an appropriate response to white matter injury in human disease.

We eliminated all activin receptor responses in the oligodendroglial lineage by conditionally knocking out the co-receptor *Acvr1b* at the OPC stage, which would prevent all downstream signaling subsequent to ligand binding to activin receptors Acvr2a or Acvr2b. By postnatal day 16, this caused a 60% reduction in the number of myelinated axons, particularly those of intermediate caliber. This finding reveals that there are activin receptor-dependent and -independent mechanisms driving axonal ensheathment with myelin membrane. This may be regulated by differential expression of activin receptor ligands across axon calibers, as experimental augmentation of axon caliber to support myelination is associated with increased expression of the activin-A subunit [28]. Myelination may also be regulated by heterogeneity of activin receptor expression in oligodendroglial lineage cells, as our data-mining of a single-cell RNA sequencing dataset [41] showed that only a subset of cells express Acvr2a at each stage of oligodendrocyte maturation.

The hypomyelination in activin receptor conditional knockout mice was associated with reduced numbers of maturing oligodendrocytes, showing the requirement for activin receptor signaling in the differentiation of a significant subset of oligodendroglial lineage cells. Importantly, activin receptor stimulation of wildtype OPCs (with activin-A) was sufficient to enhance oligodendrocyte differentiation, even in the presence of proliferation-stimulating growth factors. Although in the knockout some myelinating oligodendrocytes were still generated, these cells demonstrated impaired myelin membrane compaction, as evidenced by a persistent enlargement of the inner tongue, thinner layers of compact myelin, and myelin sheaths devoid of the compaction-inducing protein MBP. In addition, stimulation of activin receptors on wildtype maturing oligodendrocytes enhanced depolymerization of actin in membranes, a process required for myelin membrane compaction [48, 66]. Thus, we established that activin receptor signaling regulates myelination by driving oligodendrocyte differentiation and myelin membrane compaction, which to our knowledge is the first time a receptor axis has been directly linked to both processes.

These effects were found to be mediated by the ligand-binding activin receptor subtype Acvr2a, as its neutralization in wildtype cells eliminated activin-A-driven oligodendrocyte differentiation and membrane actin depolymerization. Accordingly, we showed that Acvr2a expression on oligodendroglial lineage cells in vivo coincides with oligodendrocyte differentiation and myelin generation during development and following injury. At the onset of successful remyelination, there was a concomitant downregulation of Acvr2b. Acvr2b has relatively higher affinity for activin-A compared to Acvr2a [5], and importantly its overexpression was found to impair activin-A-induced oligodendrocyte differentiation and membrane actin depolymerization. Thus, the downregulation of Acvr2b after injury may allow Acvr2a to bind ligand more readily to drive oligodendroglial lineage responses and initiate repair.

Following CNS injury, the rapid increase in expression of activin-A [1, 23, 43, 62] likely represents an attempt at repair, supported by our previous observation of increased activin-A subunit expression in focal demyelinated lesions at the onset of remyelination [45]. However, activin-A (INHBA)+ cells were found to be present in both repairing and non-repairing regions of damaged human white matter, indicating that following injury oligodendrocyte lineage cell responses are likely not regulated by activin-A, but rather by activin receptors. Indeed, regions that were actively myelinating (in perinatal tissue) or remyelinating (in multiple sclerosis lesions) demonstrated relatively higher densities of oligodendroglial lineage cells expressing Acvr2a compared to those expressing Acvr2b. However, an increase in Acvr2b+ oligodendroglial lineage cells was observed in non-repairing white matter (injured regions following perinatal brain injury, and chronic inactive multiple sclerosis lesions), which would be predicted to sequester ligand and consequently impair Acvr2a-regulated oligodendrocyte differentiation and myelin formation. Consistent with this postulate, increased levels of *Acvr2b* mRNA in umbilical cord blood at birth is associated with more severe clinical outcome in infants following hypoxic-ischemic perinatal brain injury [40].

Oligodendrocyte differentiation, myelination, and remyelination have also been associated with activation of other receptors, such as endothelin receptor B [63], RXRγ [31], and CXCR4 [50]. As we observed both activin receptor-dependent and -independent oligodendrocyte differentiation in vivo, this suggests that complementary pathways likely coordinate oligodendroglial lineage responses in parallel. This may involve distinct subsets of oligodendroglial lineage cells preferentially expressing specific receptors; indeed data-mining of a single-cell RNAseq database confirms distinct expression patterns of the abovementioned receptors within the oligodendrocyte lineage [41] (data not shown). An alternate mechanism may involve context-dependent availability of ligand; for example, we have previously demonstrated dynamic changes in activin-A protein levels during the course of remyelination [45]. Here, we provide the first evidence for a receptor axis that is dysregulated in both myelin disorders of development (perinatal brain injury) and adulthood (MS), despite having distinct etiologies. These disorders also share dysregulation of an intracellular signaling cascade, the Wnt/β-catenin pathway [20, 21], which may further antagonize Acvr2a signaling via upregulation of an inhibitor of activin-A (follistatin) [35] and a pseudo-receptor acting as a dominant negative Acvr1b (BAMBI) [59]. Whereas other members of the TGFβ superfamily (e.g. bone morphogenic proteins) have recently been shown to impair remyelination via signaling through distinct receptors (BMPR1, Acvr1a) and pathways (Smad1/5/8) [51], our data demonstrate the pro-myelination/remyelination properties of the TGFβ superfamily via activin receptor signaling. Thus, we propose that therapies specifically restoring Acvr2a-mediated signaling in oligodendroglial lineage cells could represent a novel strategy to enhance differentiation and myelin generation following CNS injury across the lifespan.

## Electronic supplementary material

Below is the link to the electronic supplementary material.

Supplementary material 1 (DOCX 9382 kb)

Supplementary material 2 (MP4 2451 kb)
