# Supplementary material for: Activin receptors regulate the oligodendrocyte lineage in health and disease
Source: Acta Neuropathol. 2018 Feb 3;135(6):887–906. doi: 10.1007/s00401-018-1813-3 (PMC5954071; doi:10.1007/s00401-018-1813-3)
Supplement: Supplementary file 1 — Supplementary material 1 (DOCX 9382 kb) [file 401_2018_1813_MOESM1_ESM.docx]

**Online Resource Supplemental Material**

**Activin receptors regulate the oligodendrocyte lineage in health and disease**

Alessandra Dillenburg, Graeme Ireland, Rebecca K. Holloway, Claire L. Davies, Frances L. Evans, Matthew Swire, Marie E. Bechler, Daniel Soong, Tracy J. Yuen, Gloria H. Su,

Julie-Clare Becher, Colin Smith, Anna Williams, Veronique E. Miron

**This PDF file includes:**

Supplemental Figures 1-9

Supplemental Tables 1-2

Caption for Supplemental Video 1

**Other Supplementary Materials for this manuscript includes the following:**

Supplemental Video 1

**Online Resource Supplemental Figures**

**Supplemental Fig.1. Conditional knockout verification.**

**
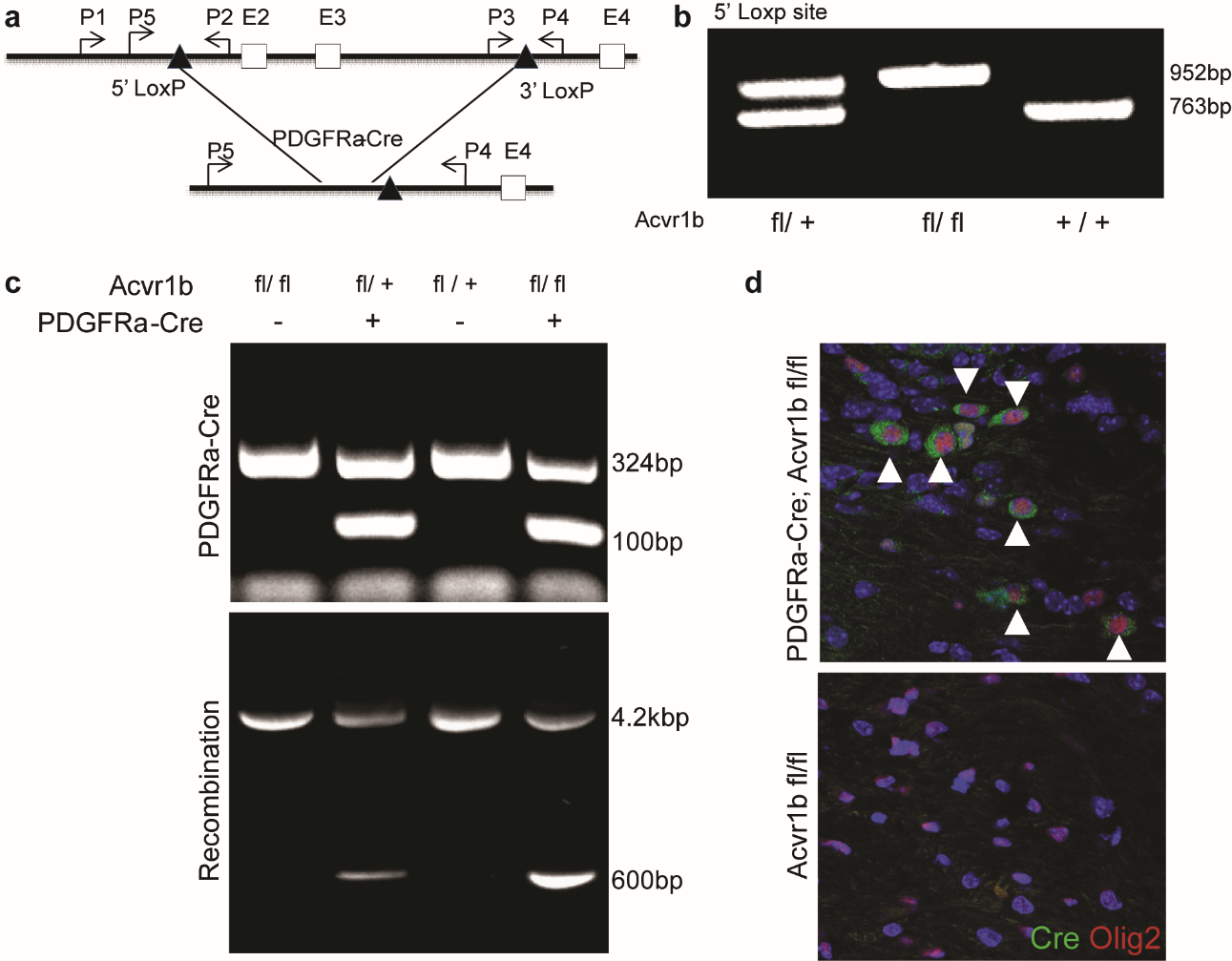
**

1. Schematic of loxP site (triangles)-floxed exons (‘E’; squares) 2 and 3 of Acvr1b allele (top) and recombined allele (bottom) and PCR-based genotyping strategies (primers (P) 1-5) for Acvr1b and Cre-mediated recombination.
2. PCR of 5’LoxP site using P1 and P2 (see Methods) in Acvr1b wildtype (+/+), heterozygotes (fl/+) and homozygotes (fl/fl), with upper bands (952 bp) representing the alleles with the LoxP site and lower band (763 bp) representing the wildtype allele.
3. Detection of the recombined fragment using P4 and P5 in Acvr1b^fl/fl^, PDGFRa-Cre;Acvr1b^fl/+^, Acvr1b^fl/+^, and PDGFRa-Cre;Acvr1b^fl/fl^ mice. Cre transgene is detected at 100 bp; 342 bp band represents IL-2 internal control. Recombined fragment (600 bp; bottom panel) in isolated total OPCs was only detected in PDGFRa-Cre+ mice (100bp; top panel); wildtype allele represented at 4.2 kbp.
4. Cre-recombinase expression (green) in oligodendrocyte lineage cells (Olig2; red) in PDGFRa-Cre; Acvr1b^fl/fl^ conditional knockouts and Acvr1b^fl/fl^ controls.

**Supplemental Fig.2. CNS hypomyelination in PDGFRa-Cre; Acvr1b^fl/fl^ mice.**

**
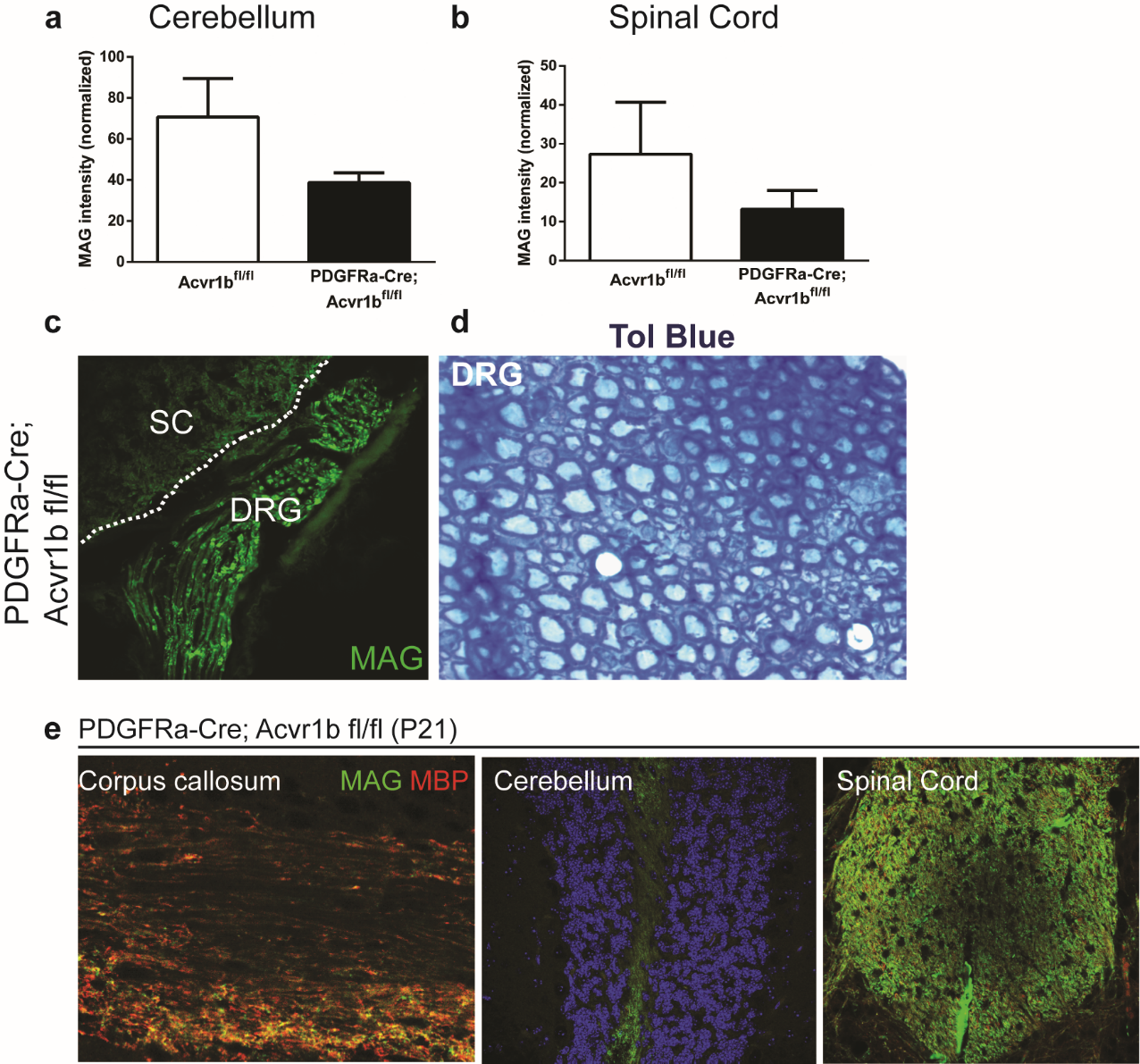
**

Mean normalized MAG intensity ± s.e.m. in the white matter of the cerebellum (a) and spinal cord (b) in P16 Acvr1b^fl/fl^ controls and PDGFRa-Cre;Acvr1b^fl/fl^ conditional knockout mice.

(c) As a control for myelin protein staining, myelin associated glycoprotein (MAG) immunostaining was still detectable in the dorsal root ganglia (DRG) attached to spinal cord (SC) of PDGFRa-Cre;ACvr1b^fl/fl^ mice, presumably Schwann cell-derived.

(d) Toluidine-blue-stained semithin resin sections of dorsal root ganglia from PDGFRa-Cre;Acvr1b^fl/fl^ mice shows normal myelination, demonstrating the CNS-specific effect of the conditional knockout on myelination.

(e) Immunofluorescent staining of MAG (green) and myelin basic protein (MBP; red) in corpus callosum, cerebellum (counterstained with Hoechst (blue)), and spinal cord in PDGFRa-Cre;Acvr1b^fl/fl^ mice at postnatal day 21, showing some myelination but hypomyelination particularly in corpus callosum and cerebellum.

**Supplemental Fig.3. Lack of oligodendroglial lineage cell death in PDGFRa-Cre; Acvr1b ^fl/fl^ mice.**

**
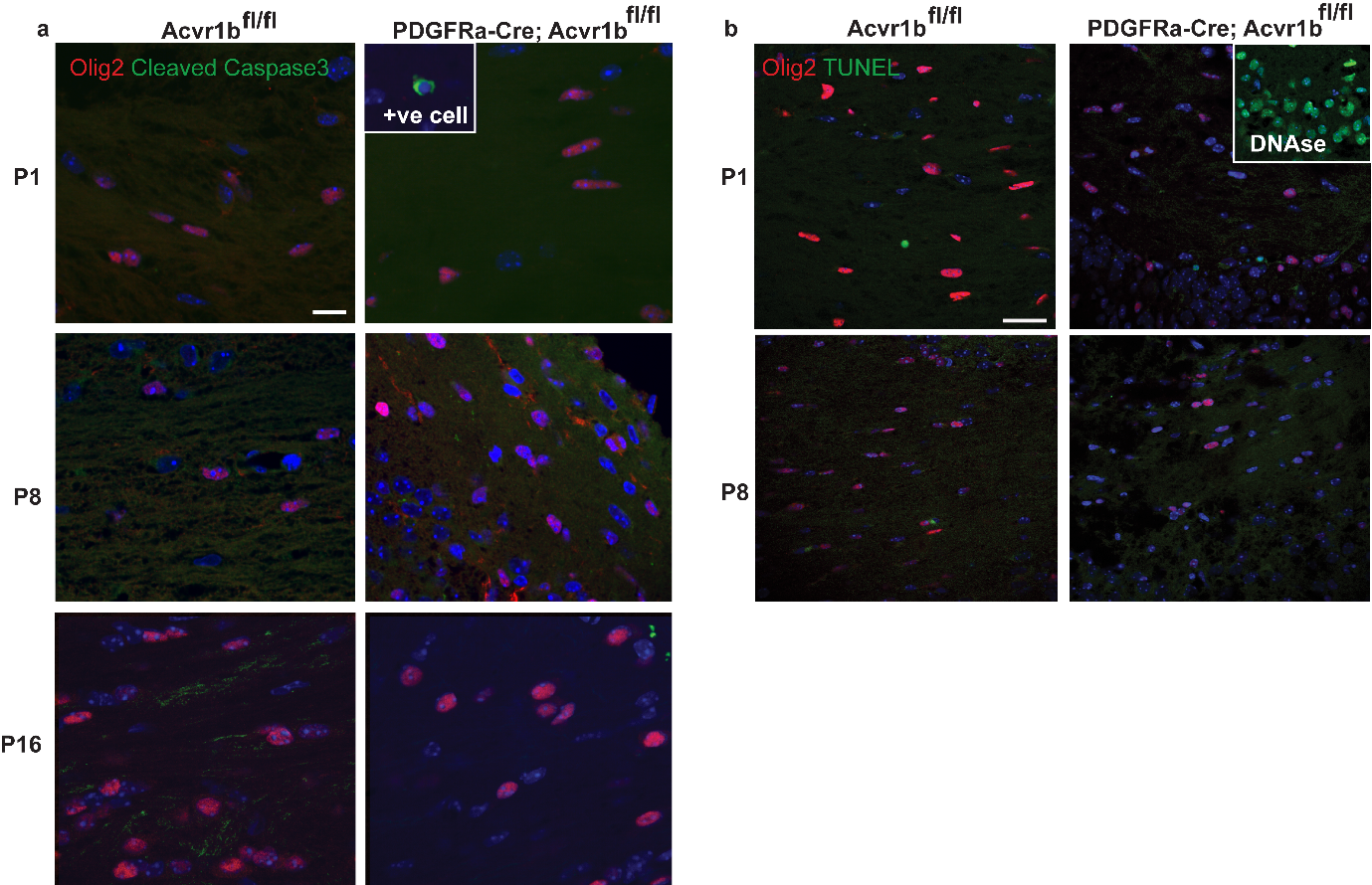
**

1. Images of corpus callosum in Acvr1b^fl/fl^ controls and PDGFRa-Cre;Acvr1b^fl/fl^ conditional knockout mice at P1, P8, and P16 stained for oligodendrocyte lineage cells (Olig2+; red) and apoptotic marker Cleaved caspase 3 (green). Inset: example of a cleaved capase-3 positive cell. Scale bar, 25 µm.
2. Images of corpus callosum in Acvr1b^fl/fl^ controls and PDGFRa-Cre;Acvr1b^fl/fl^ conditional knockout mice at P1 and P8 stained for oligodendrocyte lineage cells (Olig2+; red) and marker of double stranded breaks indicative of apoptosis (TUNEL; green). Inset: positive control DNase-I-treated section. Scale bar, 50 µm.

**Supplemental Fig.4. Expression of activin-A in developing brain.**

**
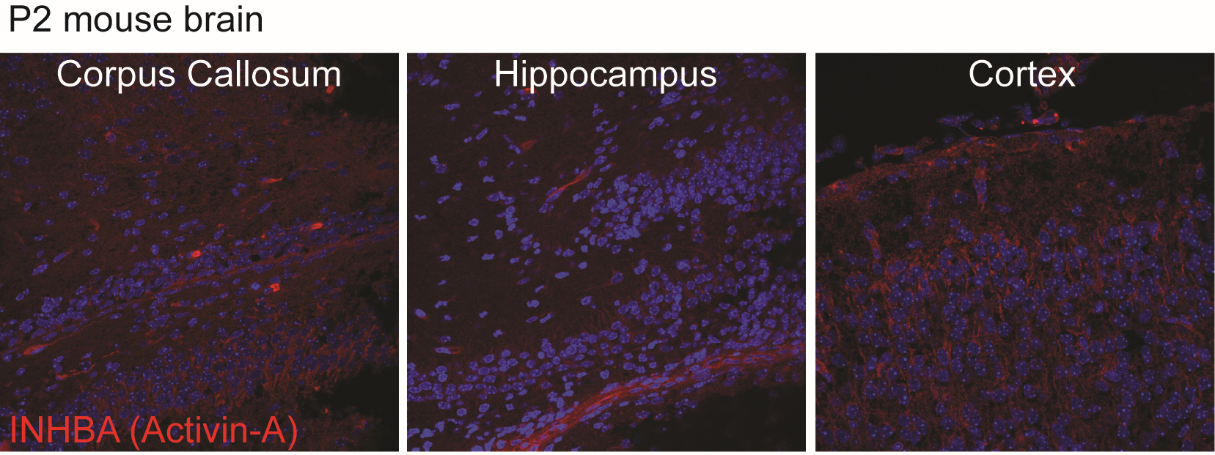
**

Immunostaining for the activin-A subunit INHBA (red) counterstained with Hoechst (blue) in postnatal day 2 (P2) sagittal sections of mouse corpus callosum, hippocampus, and cortex.

**Supplemental Fig.5. Methods for measuring axonal diameter, inner tongue thickness, and myelin thickness.**


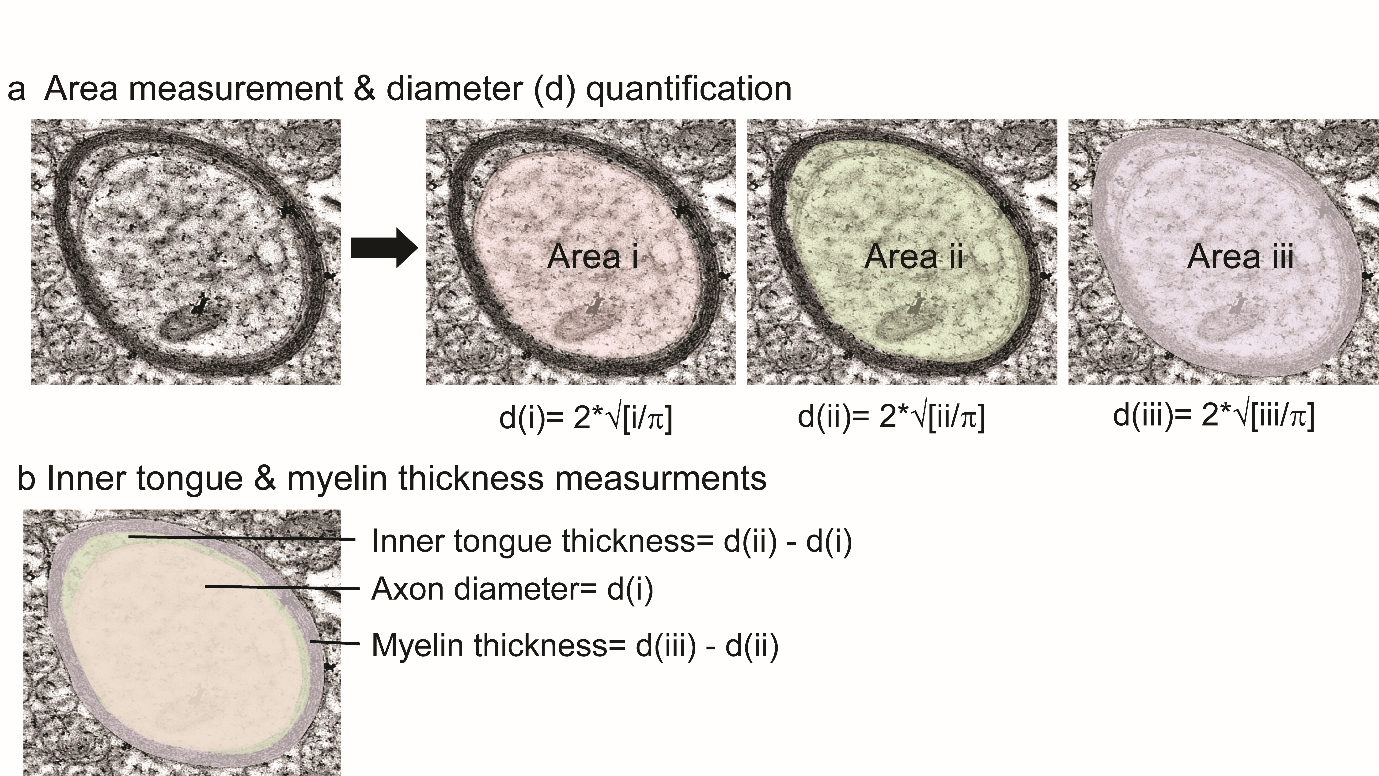


(a) Protocol for selection of area to be measured for axon (Area i), axon and inner tongue (Area ii), and axon, inner tongue, and compact myelin (Area iii). Diameter was calculated from the area as indicated.

(b) Equations for determining thickness of inner tongue and myelin based on diameter calculations.

**Supplemental Fig.6. No effect of activin-A on mature oligodendrocyte membrane size and myelin sheath number/ length.**

**
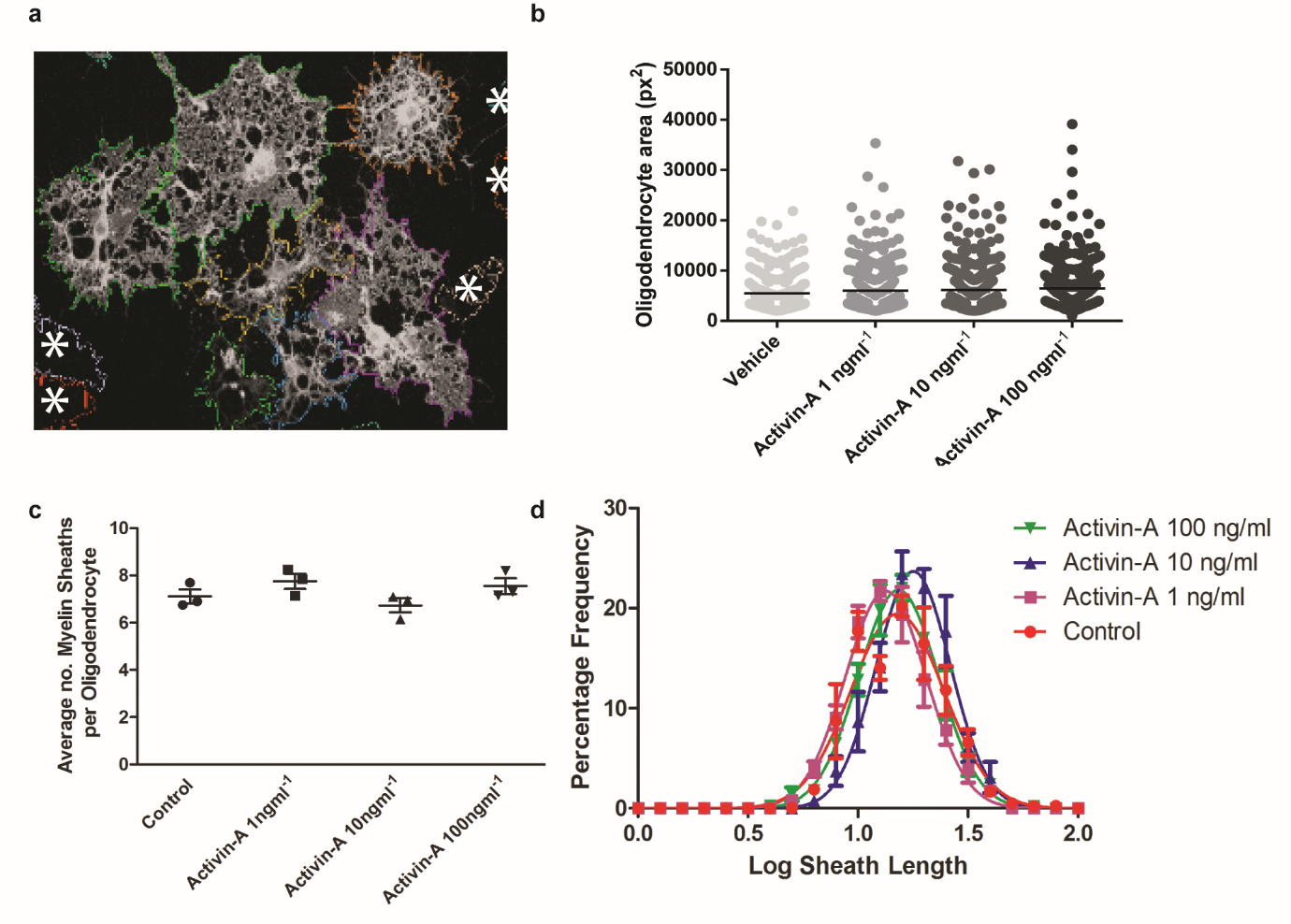
**

1. Representative image of automated delineation of individual oligodendrocyte membranes/processes using Columbus software. False positives (<2000 px^2^; asterisks) were removed from analysis.
2. Oligodendrocyte area (px^2^) following 3-day treatment with activin-A (1-100 ngml^-1^). Mean oligodendrocyte area per experiment was not significantly different between groups, *n*=3 biological replicates.
3. Average number of myelin sheaths per oligodendrocyte ± s.e.m. formed on microfibers following treatment with activin-A (1-100 ngml^-1^) or vehicle control. *n*=3 biological replicates.
4. Log length of myelin sheaths formed on microfibers following treatment with activin-A (1-100 ngml^-1^) or vehicle control. *n*=3 biological replicates.

**Supplemental Fig.7. Positive control for activin receptor antibodies and activin receptor subtype mRNA expression during brain development.**

**
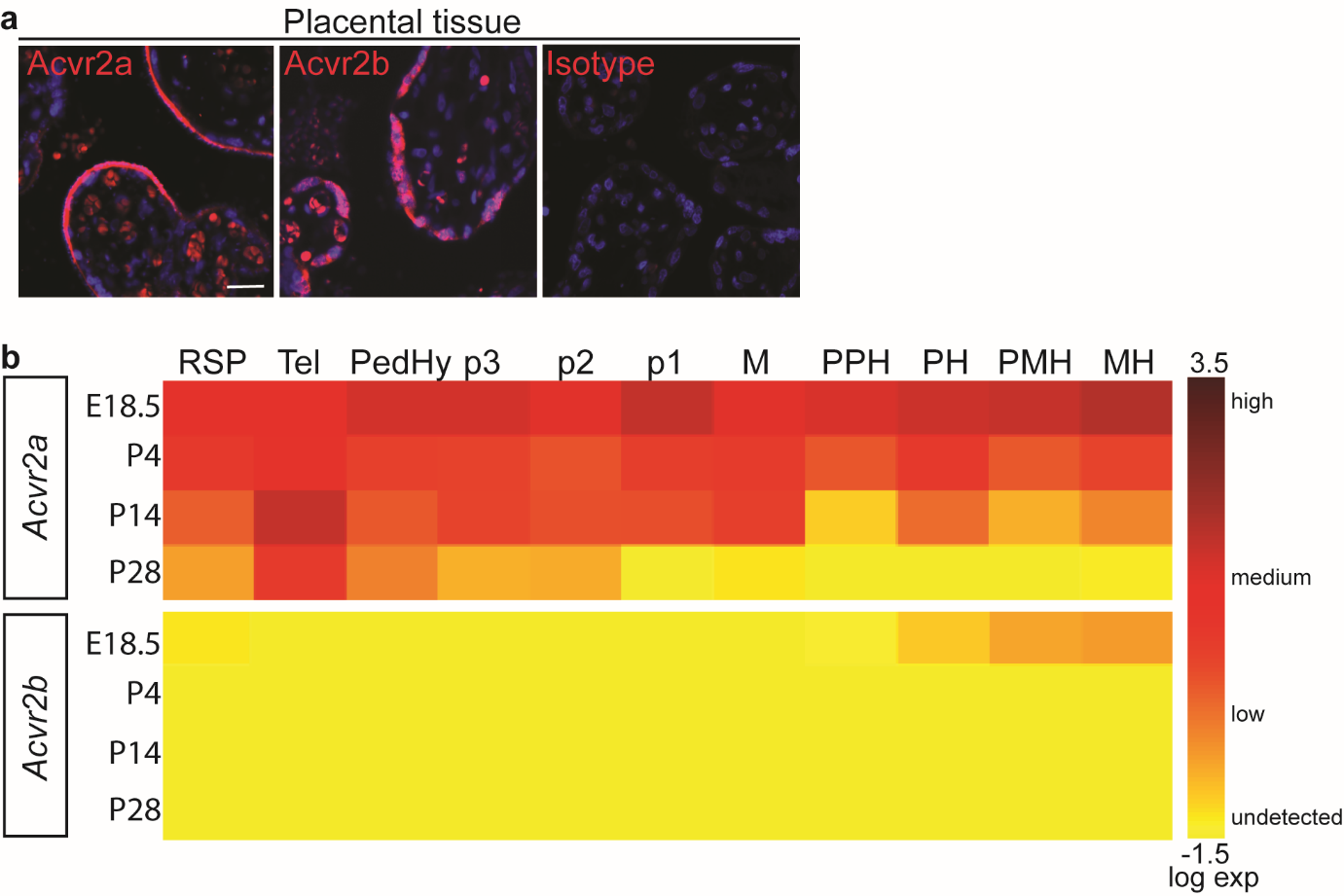
**

1. Positive control for activin receptor expression: placental tissue was stained with antibodies against Acvr2a (left), Acvr2b (middle) or isotype control (right) and counterstained with Hoechst (blue). Scale bar, 20 μm.
2. Heatmaps of log-transformed mRNA expression levels for *Acvr2a* (A) and *Acvr2b* (B) detected by *in situ* hybridization, generated in the Allen Developing Mouse Brain Atlas (*developingmouse.brain-map.org*), prior to onset of myelination (embryonic day (E) 18.5) up to when myelination is mostly complete (postnatal day (P) 28). Brain areas investigated are rostral secondary prosencephalon (RSP), telencephalic vesicle (Tel), peduncular (caudal) hypothalamus (PedHy), prosomere 1-3 (p1-3), midbrain (M), prepontine hindbrain (PPH), pontine hindbrain (PH), pontemedullary hindbrain (PMH), medullary hindbrain (MH). Yellow indicates undetected, orange low detection, red medium expression and burgundy high expression.

**Supplemental Fig.8. Neutralization of Acvr2b in oligodendroglial lineage cells.**

**
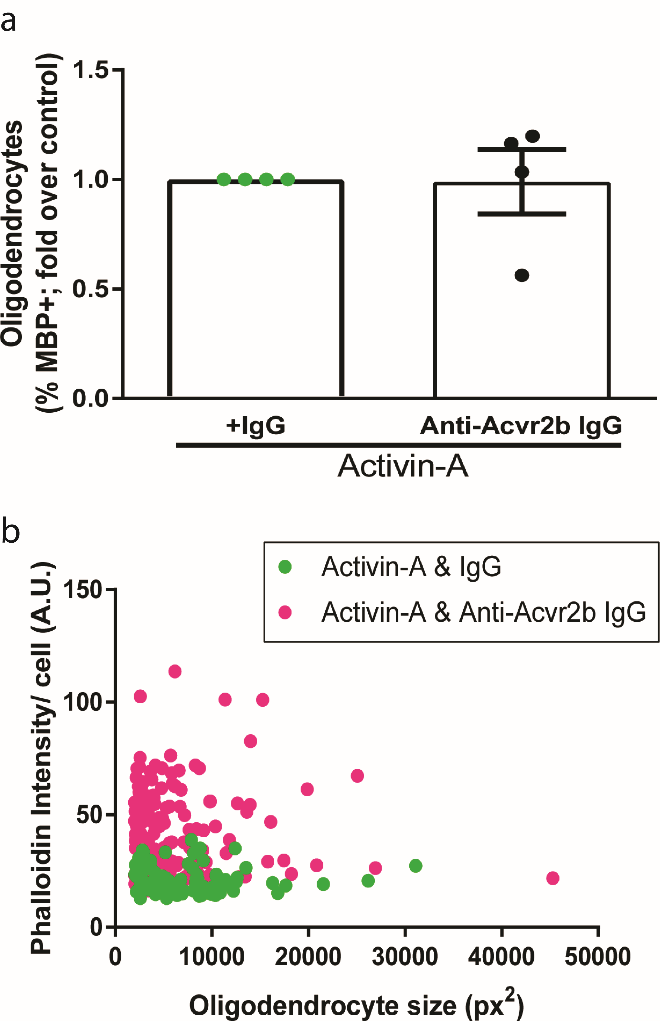
**

1. Quantification of percentage of mature oligodendrocytes (MBP+) in cultures treated with activin-A (10 ng/ml) and either IgG control or anti-Acvr2b neutralizing antibody. *n*=4 biological replicates.
2. Phalloidin intensity per MBP+ cell versus oligodendrocyte size, in maturing oligodendrocyte cultures treated with activin-A (10 ng/ml) and either IgG control (green) or anti-Acvr2b neutralizing antibody (magenta).

**Supplemental Fig.9. Pathological assessment of human brain tissue.**

**
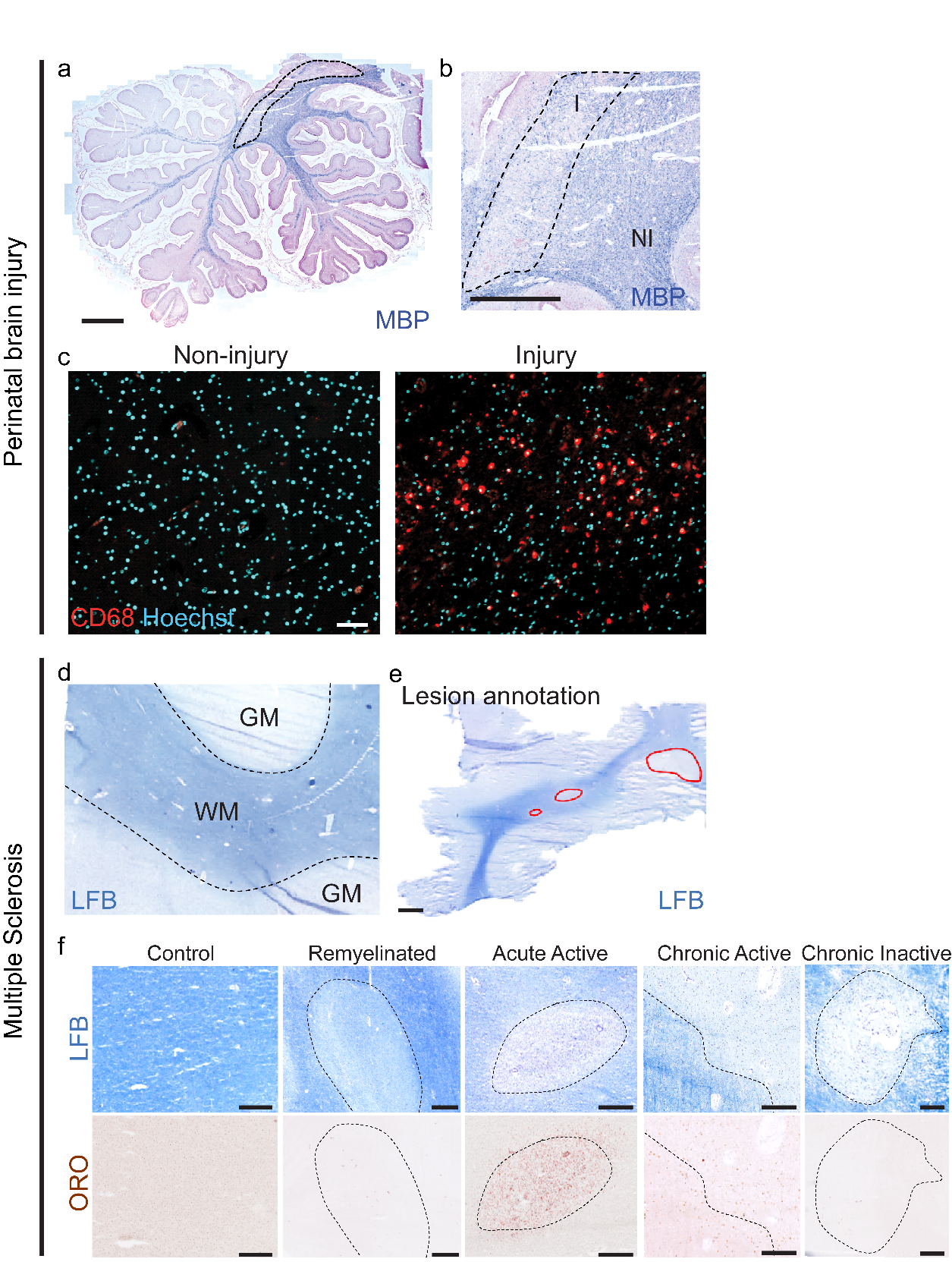
**

Perinatal brain injury tissue pathological assessment (a-c).

(a) Image of human infant cerebellum with an absence of myelin basic protein (MBP) within injured area (dotted line). Scale bar, 2000 µm.

(b) Higher magnification of injured area (I) shown in (a), compared to adjacent non-injured area (NI) which shows normal myelination (MBP; blue). Scale bar, 1000 µm.

(c) Higher densities of activated microglia/ macrophages (CD68+; red) were observed in injured areas versus non-injured areas. Counterstain Hoechst (turquoise). Scale bar, 50 µm.

Multiple sclerosis (MS) brain lesion pathological assessment (d-f).

(d) Luxol Fast Blue (LFB; blue) staining was used to clearly delineate white matter (WM) from grey matter (GM). Scale bar, 500 µm.

(e) MS lesions were digitally annotated (red circles) in Zen software to ensure that the same area was quantified for various readouts. Scale bar, 2000 µm.

(f) MS lesions were pathologically characterized according to the International Classification of Neurological Diseases, using LFB to stain intact myelin (blue) and Oil Red O (ORO) to stain myelin debris (brown). Healthy control tissue shows intact myelin and no myelin debris. Fully remyelinated lesions show intermediate intensity of LFB (‘shadow plaque’) with little to no myelin debris. Acute active lesions show a diffuse LFB border with demyelination and an abundance of myelin debris throughout the lesion. Chronic Active lesions (rim) show loss of LFB and some myelin debris. Chronic Inactive lesions show significant demyelination and no myelin debris. Scale bar, 500 µm.

**Supplementary Tables**

**Table 1: Post-mortem Perinatal Brain Injury Tissue.**

Information on source of perinatal brain injury tissue showing white matter damage, used for analysis.

| **Case** | **Sex** | **Gestational Age (wks)** | **Survival (days)** | **Neuropathological information** |
| --- | --- | --- | --- | --- |
| **1** | F | 33 | 0 | Intrauterine growth restriction, established hypoxia, ischemia |
| **2** | M | 38 | <1 | Gliosis, established and recent hypoxia, cell death |
| **3** | M | 24 | 0 | Gliosis, recent hypoxia, cell death |
| **4** | F | 41 | 4 | Recent hypoxia, gliosis, perivascular foci in white matter |
| **5**  **(2 blocks)** | M | 32 | <30 | White matter gliosis, macrophage accumulation |

**Table 2. Post-mortem Multiple Sclerosis Lesion Tissue**

Information on source of human adult white matter brain sections in multiple sclerosis patients or controls, used for analysis.

|  | **Classification** | **Sex** | | **Age** | **Disease duration (yrs)** | | **Block** | **Lesions Analyzed** | | | |
| --- | --- | --- | --- | --- | --- | --- | --- | --- | --- | --- | --- |
|  |  |  |  |  |  |  |  | **Active** | **Chronic Active (rim)** | **Chronic Inactive** | **Remyel-inated** |
| **MS Cases** | SPMS | M | | 44 | 10 | | 1 | 1 | 0 | 2 | 0 |
|  | SPMS | M | | 40 | 9 | | 1 | 1 | 0 | 1 | 3 |
|  |  |  |  |  |  |  | 2 | 1 | 3 | 2 | 2 |
|  | PPMS | M | | 37 | 27 | | 1 | 0 | 1 | 1 | 5 |
|  | SPMS | F | | 57 | 27 | | 1 | 0 | 0 | 0 | 4 |
|  | SPMS | F | | 46 | 25 | | 1 | 0 | 1 | 1 | 5 |
|  |  |  |  |  |  |  | 2 | 0 | 2 | 0 | 0 |
|  | SPMS | F | | 42 | 19 | | 1 | 2 | 0 | 0 | 2 |
|  | SPMS | F | | 49 | 14 | | 1 | 2 | 1 | 0 | 0 |
|  | SPMS | F | | 57 | 19 | | 1 | 0 | 2 | 1 | 2 |
| **TOTAL** | | | | | | | | **7** | **10** | **8** | **23** |
| **Controls** | Carcinoma of the tongue | | M | 35 | - | 1 | | - | - | - | - |
|  | Myelodys-plastic syndrome, RA | | M | 82 | - | 1 | | - | - | - | - |
|  | Cardiac failure | | M | 64 | - | 1 | | - | - | - | - |
|  | Ovarian cancer | | F | 60 | - | 1 | | - | - | - | - |

**Supplemental Video 1 caption**

(0-10 seconds) Compared to heterozygous littermate (PDGFRa-Cre;Acvr1b^fl/+^; left), homozygous conditional knockout of activin receptor signalling in OPCs (PDGFRa-Cre; Acvr1b^fl/fl^; right) shows hunched posture and tremor.

(10-14 seconds) Homozygous conditional knockout mouse showing tremor.

(14-20 seconds) Homozygous conditional knockout mouse showing stiff tail with tremor.
